# Shared parameter modeling of longitudinal data allowing for possibly informative visiting process and terminal event

**DOI:** 10.1093/biostatistics/kxae041

**Published:** 2024-10-23

**Authors:** Christos Thomadakis, Loukia Meligkotsidou, Nikos Pantazis, Giota Touloumi

**Affiliations:** Department of Hygiene, Epidemiology and Medical Statistics, Medical School, National and Kapodistrian University of Athens, Mikras Asias 75, Athens, 115 27, Greece; Department of Mathematics, National and Kapodistrian University of Athens, Panepistemiopolis, Athens, 157 84, Greece; Department of Mathematics, National and Kapodistrian University of Athens, Panepistemiopolis, Athens, 157 84, Greece; Department of Hygiene, Epidemiology and Medical Statistics, Medical School, National and Kapodistrian University of Athens, Mikras Asias 75, Athens, 115 27, Greece; Department of Hygiene, Epidemiology and Medical Statistics, Medical School, National and Kapodistrian University of Athens, Mikras Asias 75, Athens, 115 27, Greece

**Keywords:** informative visiting, joint modeling, linear mixed model, observation process, shared parameter models, visiting process

## Abstract

Joint modeling of longitudinal and time-to-event data, particularly through shared parameter models (SPMs), is a common approach for handling longitudinal marker data with an informative terminal event. A critical but often neglected assumption in this context is that the visiting/observation process is noninformative, depending solely on past marker values and visit times. When this assumption fails, the visiting process becomes informative, resulting potentially to biased SPM estimates. Existing methods generally rely on a conditional independence assumption, positing that the marker model, visiting process, and time-to-event model are independent given shared or correlated random effects. Moreover, they are typically built on an intensity-based visiting process using calendar time. This study introduces a unified approach for jointly modeling a normally distributed marker, the visiting process, and time-to-event data in the form of competing risks. Our model conditions on the history of observed marker values, prior visit times, the marker’s random effects, and possibly a frailty term independent of the random effects. While our approach aligns with the shared-parameter framework, it does not presume conditional independence between the processes. Additionally, the visiting process can be defined on either a gap time scale, via proportional hazard models, or a calendar time scale, via proportional intensity models. Through extensive simulation studies, we assess the performance of our proposed methodology. We demonstrate that disregarding an informative visiting process can yield significantly biased marker estimates. However, misspecification of the visiting process can also lead to biased estimates. The gap time formulation exhibits greater robustness compared to the intensity-based model when the visiting process is misspecified. In general, enriching the visiting process with prior visit history enhances performance. We further apply our methodology to real longitudinal data from HIV, where visit frequency varies substantially among individuals.

## 1 Introduction

Nowadays, electronic health records (EHRs) are being increasingly used to address complex clinical questions, which often require longitudinal measurements of a marker related to some disease. However, many methodological challenges have been posed, with one issue being that the timing at which the marker is measured may be related to disease severity. Thus, patients with worse/better health conditions may contact clinics more/less frequently, which can lead to biased estimates of the marker trends when using standard methods (Pullenayegum and Lim 2016), e.g. linear mixed models (LMMs). In our motivating example from the epidemiology of HIV infection, EHR longitudinal data from the Athens Multicenter AIDS Cohort Study (AMACS) are available, with special focus on the number of CD4 cells, an immunological marker of HIV disease progression. Descriptive inspection of the data revealed that the frequency of measurements differs substantially among individuals, which questions the validity of standard analyses, as visits can be individual-driven. Another issue is that, if an individual dies or disengages from care (competing risks), CD4 cell count measurements are no longer feasible.

Although there is a substantial body of literature on longitudinal data with monotone and non-monotone missingness (e.g. Troxel et al. 1998), these methods are not generally applicable to irregularly measured longitudinal data. With regular data, there is a fixed set of discrete time points, whereas for irregular data such a set does not exist by definition. Hence, standard methods, such as multiple imputation, are infeasible, as they would require an infinitely large number of missing data indicators. Thus, several authors (e.g. Lin and Ying 2001, 2003) have used counting processes to model arbitrary visit times. Although such methods for handling irregularly observed longitudinal data are less well studied than methods for repeated measures with monotone missingness, they have recently received some attention.

Most missing data methods can be characterized in terms of the standard taxonomy by Rubin (1976), ie missing completely at random (MCAR), missing at random (MAR), and missing not at random (MNAR). Pullenayegum and Lim (2016) similarly characterized the visiting process through analogous definitions. Visiting is completely at random (VCAR) when visit times and marker values are independent, implying that all standard methods ignoring the visiting process (e.g. GEE and LMMs) lead to unbiased estimates. Visiting is at random (VAR) when, given data recorded up to time *t*, visiting at *t* is independent of marker values at *t*. It has been shown that likelihood-based methods ignoring a VCAR or VAR mechanism provide correct inference if correctly specified (Lipsitz et al. 2002). Finally, visiting is not at random (VNAR) when, conditionally on data recorded up to time *t*, visiting at *t* is not independent of marker values at *t*. In such cases, the visiting process should be jointly modeled to obtain unbiased estimates for the marker model. Most VNAR models are semiparametric, extending the model by Lin and Ying (2001) by assuming that, conditionally on subject-specific random effects that are shared or correlated between the marker process and the visiting process, the two processes are independent (e.g. Huang et al. 2006; Liang et al. 2009). Marginally (integrating out the random effects), though, this induces dependence between visiting and unobserved marker values.

In most real data applications, there may also be a single or multiple terminal events (e.g. death and disengagement from care) that might informatively censor the longitudinal marker, leading to MNAR missingness. To obtain unbiased estimates in such settings, joint modeling of marker evolution and time to event is needed. Fewer approaches exist for jointly modeling (i) the marker evolution, (ii) an VNAR visiting process, and (iii) an informative (MNAR) time to event. Most authors postulated semiparametric models (e.g. Sun et al. 2007; He et al. 2009; Sun et al. 2012; Su and Jiang 2018; Yu et al. 2019), typically leaving the distribution of the latent variables unspecified or using nonparametric intercepts in the marker model, whereas Liu et al. (2008) proposed a fully parametric joint model.

Although the aforementioned semiparametric approaches may include some nonparametric components, they rely on a conditional independence assumption. As pointed out in Pullenayegum and Lim (2016), assessment of this assumption has received little attention despite its potential importance. On top of that, it has been recently shown that violating the conditional independence assumption in longitudinal and time-to-event joint models can lead to seriously biased marker estimates (Thomadakis et al. 2019). Another issue is that the above approaches model only the intensity of the visiting process as a function of time since baseline, *t* (calendar time models). However, modeling the hazard of the time since most recent visit would be statistically sound as well (Cook and Lawless 2007); yet it can lead to a better fit. To our knowledge, there is no literature in the analysis of repeated measures accounting for both informative (VNAR) visiting process and informative (MNAR) competing risks.

In this article, we propose a unified approach to model the evolution of a longitudinal marker, the visiting process, and the time-to-event process in the form of competing risks. The proposed approach relaxes the conditional independence assumption between the processes and can adopt either a gap time or calendar time model for the visiting mechanism. In Section 2, we introduce the proposed model and describe the estimating procedure. In Section 3, an extensive simulation study is carried out to evaluate the performance of the proposed model when correctly specified and when certain features of it are misspecified. In Section 4, we apply the proposed methodology to EHR data from the AMACS study. Concluding remarks are presented in Section 5, along with a discussion of the limitations and possible extensions of our approach.

## 2 Proposed Model

### 2.1 Longitudinal marker model

In this subsection, we describe the longitudinal marker process of our approach. Specifically, to model the marginal distribution of the marker values, we assume a standard LMM of the form $Y_i (t) = \boldsymbol {X}_i^{\mathrm{ \top }} (t)\boldsymbol {\beta } + \boldsymbol {Z}_i^{\mathrm{ \top }} (t)\boldsymbol {b}_i + {\mathrm{ \epsilon }}_i (t)$, where $Y_i (t)$ denotes the observed marker value at time *t* since baseline, $\boldsymbol {X}_i (t)$ and $\boldsymbol {Z}_i (t)$ denote the fixed- and random-effect design matrices at *t*, and $\boldsymbol {\beta }$ and $\boldsymbol {b}_i \sim N({\bf 0}, \boldsymbol {D})$ are the fixed and random effects, respectively, with ${\mathrm{ \epsilon }}_i (t)\sim N(0, \sigma ^2 )$. As commonly interpreted in the literature, $m_i (t) = \boldsymbol {X}_i^{\mathrm{ \top }} (t)\boldsymbol {\beta } + \boldsymbol {Z}_i^{\mathrm{ \top }} (t)\boldsymbol {b}_i$ denotes the “true” marker value at *t* (removing random fluctuation). We assume that the marker for individual *i* has been evaluated at visit times $0 = t_{i1} < t_{i2} < \ldots < t_{n_i }$, where *n_i_* is the observed number of visits for individual *i*. The observed marker values are denoted by $\boldsymbol {Y}_i^{\mathrm{ \top }} = (Y_{i1} , Y_{i2} , \ldots , Y_{in_i } )$, and, defined similarly, $\boldsymbol {X}_i$ and $\boldsymbol {Z}_i$ denote the observed fixed- and random-effect design matrices, respectively. The marker model parameters are then defined as $\boldsymbol {\theta }_l^{\mathrm{ \top }} = (\boldsymbol {\beta }^{\mathrm{ \top }} , {\mathop{\rm vech}\nolimits} (\boldsymbol {D})^{\mathrm{ \top }} , \sigma ^2 )$, where ${\mathop{\rm vech}\nolimits}$ is the “vector-half” operator stacking the columns of the lower triangular part of a symmetric matrix.

### 2.2 Visiting process

Rather than assuming that the visit times are fixed, or at least their distribution is noninformative and thus ignorable (Pullenayegum and Lim 2016), we assume that $\{ t_{ij} , j = 1, 2, \ldots , n_i \}$ is a realization of some stochastic process that carries information which cannot be ignored. The visiting process can be uniquely characterized by the counting process, $N_i^{\mathrm{ \star }} (t)$, which records the total number of visits up to *t*. It is also assumed that the marker data are subject to mutually exclusive events in the form of competing risks, with $T_i^{\mathrm{ \star }}$ denoting the time to the first occurring event, $K_i \in \{ 1, 2, \ldots , K\}$ the corresponding event type, and *C_i_* a noninformative right censoring time. Thus, the follow-up time for individual *i* is denoted by $T_i = \min (T_i^{\mathrm{ \star }} , C_i )$, where $T_i > t_{in_i }$. Similarly, the observable counting process for the visits is denoted by $N_i (t) = N_i^{\mathrm{ \star }} \{ \min (t, T_i )\}$.

#### 2.2.1 Gap time model

**Fig. 1:**
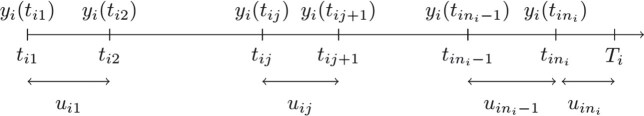
Schematic representation of marker values, visit times, and times between visits.

To model the visiting process, we first specify a model for the gap times between visits, $u_{ij} = t_{ij + 1} - t_{ij} , j = 1, 2, \ldots , n_i - 1$, and $u_{in_i } = T_i - t_{in_i }$, where $u_{ij} , j = 1, \ldots , n_i - 1$ are uncensored times, whereas $u_{in_i }$ is a right-censored time. This is schematically presented in Fig. 1. We consider proportional hazard models, e.g. for the *j*th gap time we assume

(2.1) $$\eqalign{ & h_{vj} \left\{ {u|\overline {\boldsymbol {X}} _{vi} (t_{ij} ), \bar Y_i (t_{ij} ), {\boldsymbol {b}}_i ;{\boldsymbol {\theta }}_v , {\boldsymbol {\beta }}} \right\} \cr & \quad = h_{v0} (u)\exp [{\boldsymbol {\gamma }}_v^{\mathrm{ \top }} \overline {\boldsymbol {X}} _{vi} (t_{ij} ) + {\boldsymbol {\phi }}_v^{\mathrm{ \top }} \boldsymbol {g}_v \{ \bar Y_i (t_{ij} )\} + \alpha _{v1} m_i (0) + \alpha _{v2} m_i^\prime (t_{ij} + u)], \cr}$$

where *u* denotes the time since the most recent visit and $t_{ij} + u$ is the current time since baseline. Moreover, $\overline {\boldsymbol {X}} _{vi} (t_{ij} )$ and $\bar Y_i (t_{ij} )$ denote fixed dimension time-dependent covariates that are derived from histories of all observed values of visits and marker values up to *t_ij_*, respectively, where $\boldsymbol {g}_v$ can be any vector-valued function. For example, $\overline {\boldsymbol {X}} _{vi} (t_{ij} )$ and $\bar Y_i (t_{ij} )$ can include the current number of visits (just prior to *t*), $N_i^{\mathrm{ \star }} (t^ - )$, and the most recent observed marker value, *Y_ij_*, respectively. Furthermore, $m_i (0)$ denotes the “true” marker value at baseline and $m_i^\prime (t) = {{\partial \boldsymbol {X}_i^{\mathrm{ \top }} (t)} \over {\partial t}}\boldsymbol {\beta } + {{\partial \boldsymbol {Z}_i^{\mathrm{ \top }} (t)} \over {\partial t}}\boldsymbol {b}_i$ the “true” marker rate of change (slope) at time since baseline, *t*. To model the baseline hazard level, we assumed $h_{v0} (u) = \exp \left\{ {\boldsymbol {B}_v^{\mathrm{ \top }} (u)\boldsymbol {\psi }_v } \right\}$, where $\boldsymbol {B}_v$ is a cubic B-spline basis at *u*. In model (2.1), it is implicitly assumed that visiting at current time *t* is independent of marker values at or after time *t* conditional on previously observed marker values and visits, and the random effects. The parameters of the visiting model are denoted by $\boldsymbol {\theta }_v^{\mathrm{ \top }} = (\boldsymbol {\psi }_v^{\mathrm{ \top }} , \boldsymbol {\gamma }_v^{\mathrm{ \top }} , {\boldsymbol {\phi }}_v^{\mathrm{ \top }} , \alpha _{v1} , \alpha _{v2} )$.

Our model lies within the SPM framework, but, in contrast to most approaches that rely on a conditional independence assumption, we propose a model that conditions on both the observed marker data and the random effects. If both $\alpha _{v1} = \alpha _{v2} = 0$, the model suggests VCAR (if $\boldsymbol {\gamma }_v = {\boldsymbol {\phi }}_v = {\bf 0}$) or VAR (if $\boldsymbol {\gamma }_v \ne {\bf 0}$ or ${\boldsymbol {\phi }}_v \ne {\bf 0}$) for the visiting process. However, if either $\alpha _{v1} \ne 0$ or $\alpha _{v2} \ne 0$, the process is VNAR, as visiting depends on the unobserved random effects, thereby making the visiting process informative (McCulloch et al. 2016). Such a situation could occur when (i) individuals with steeper or less steep latent slopes tend to make visits more frequently or (ii) a mixture of scheduled (thus non-informative) and individual-initiated (possibly symptom-driven) visit times exists. However, as is always the case with missing data approaches, the above statements require a correctly specified model (Molenberghs et al. 2008). Recall that potential dependence between the gap times of the same individual can be accounted for, e.g. by including the previous gap time, $u_{ij - 1}$, in $\overline {\boldsymbol {X}} _{vi} (t_{ij} )$ (for *j* > 1). With all covariates being constant, it is assumed that the hazard of making a visit varies only with time since the most recent visit. However, a calendar/chronological trend in gap times can also be taken into account, at least approximately, by including the *j*th visit time *t_ij_* in $\overline {\boldsymbol {X}} _{vi} (t_{ij} )$, where $t_{ij} = \sum\limits_{k = 1}^{j - 1} {u_{ik} }$.

#### 2.2.2 Intensity-based model using calendar time

Similarly to most approaches in the literature, the visiting process can be modeled in terms of the intensity of the counting process, $N_i^{\mathrm{ \star }} (t)$. Thus, assuming that $t_{ij} \le t < t_{ij + 1}$, a similar model can be postulated as follows

(2.2) \begin{align*}
&E\left\{d{ N_{i}^{\star} }(t)|{ \overline{ \boldsymbol{X} }_{vi} }(t_{ij}),{ \overline{Y}_{i} }(t_{ij}),{ { \boldsymbol{b} }_{i} };{ { \boldsymbol{\theta} }_{v} },{ \boldsymbol{\beta} }\right\} \nonumber \\
&\quad =\lambda_{v0}(t)
\exp \Big[
{ \boldsymbol{\gamma} }_{v}^{\top}{ \overline{ \boldsymbol{X} }_{vi} }(t_{ij}) + { \boldsymbol{\phi} }_{v}^{\top}{ \boldsymbol{g} }_{v}\{{ \overline{Y}_{i} }(t_{ij})\} + \alpha_{v1}m_{i}(0)+
\alpha_{v2}m_{i}^{\prime}(t)
\Big], \label{eq:CalTime}
\end{align*}

where $E\left\{ {dN_i^{\mathrm{ \star }} (t)} \right\}$ is the intensity function, ie the instantaneous probability of a visit occurring at time since baseline *t*, conditional on the process history, with $dN_i^{\mathrm{ \star }} (t) = N_i^{\mathrm{ \star }} (t) - N_i^{\mathrm{ \star }} (t^ - )$ denoting an indicator function of making a visit at *t*. All remaining terms have the same meaning as in Equation (2.1), but $\lambda _{v0} (t) = \exp \left\{ {\boldsymbol {B}_v^{\mathrm{ \top }} (t)\boldsymbol {\psi }_v } \right\}$ denotes the baseline intensity function modeled through cubic B-splines of time since baseline. The interpretation of the parameters $\boldsymbol {\gamma }_v , \;{\boldsymbol {\phi }}_v , \;\alpha _{v1}$, and $\alpha _{v2}$, in terms of characterizing the visiting process (VCAR, VAR, and VNAR), remains the same as in subsection 2.2.1. Under this model, the probability of making a visit does not vary with the time since most recent visit, $t - t_{ij}$. However, prior visit times, such as $u_{ij - 1}$, or the total number of visits so far, $N_i^{\mathrm{ \star }} (t^ - )$, can be included in $\overline {\boldsymbol {X}} _{vi} (t_{ij} )$. Similar to the gap-time model, visiting at current time *t* is assumed to be independent of future and current marker values, conditional on previously observed marker values and the random effects.

#### 2.2.3 Modeling remaining individual-level heterogeneity using frailties

Our approach, described in subsections 2.2.1 and 2.2.2, accounts for remaining individual-level heterogeneity in the visiting process by utilizing functions of previously observed visits, as well as the observed marker values and the marker random effects. However, in some cases, this may not suffice. To account for additional unexplained heterogeneity in the visiting process, we extended our previously described approach by including a frailty term, unrelated to the marker random effects. Formally, we incorporate a frailty term, $w_i \sim {\mathop{\rm Gamma}\nolimits} (1/\eta , 1/\eta )$, in Equations (2.1) and (2.2), where $E(w_i ) = 1$ and $Var(w_i ) = \eta$. The level of remaining heterogeneity is represented by the value of *η*: ie $\eta \approx 0$ suggests no remaining heterogeneity, whereas a large value of *η* indicates substantial remaining individual-level heterogeneity. In this setting, $\boldsymbol {\theta }_v^{\mathrm{ \top }} = (\boldsymbol {\psi }_v^{\mathrm{ \top }} , \boldsymbol {\gamma }_v^{\mathrm{ \top }} , {\boldsymbol {\phi }}_v^{\mathrm{ \top }} , \alpha _{v1} , \alpha _{v2} , \eta )$.

### 2.3 Competing risks

To model the competing risks, we specify a similar model for each cause-specific hazard function conditional on the observed marker values, other possibly time-dependent covariates, and the random effects. Assuming that $t_{ij} \le t < t_{ij + 1}$, the *k*th cause-specific hazard function is equal to

(2.3) $$\eqalign{ & h_k \left\{ {t|\overline {\boldsymbol {X}} _{ski} (t_{ij} ), \bar Y_i (t_{ij} ), \boldsymbol {b}_i ;{\boldsymbol {\theta }}_{sk} , {\boldsymbol {\beta }}} \right\} \cr & \quad = h_{sk0} (t)\exp [\boldsymbol {\gamma }_{sk}^{\mathrm{ \top }} \overline {\boldsymbol {X}} _{ski} (t_{ij} ) + {\boldsymbol {\phi }}_{sk}^{\mathrm{ \top }} \boldsymbol {g}_{sk} \{ \bar Y_i (t_{ij} )\} + \alpha _{sk1} m_i (0) + \alpha _{sk2} m_i^\prime (t)], \cr}$$

where $\overline {\boldsymbol {X}} _{ski} (t_{ij} )$ and $\bar Y_i (t_{ij} )$ denote the histories of observed values of time-dependent covariates (which can be cause-specific) and marker values up to *t_ij_*, respectively. It should be noted that $\overline {\boldsymbol {X}} _{ski} ()$ may be different from $\overline {\boldsymbol {X}} _{vi} ()$; In fact, $\overline {\boldsymbol {X}} _{ski} ()$ may include previous visit times. For example, if one of the competing risks is disengagement from care, it may be reasonable to assume that the risk for disengagement from care at time since baseline, *t*, depends on prior visit times (before *t*). Moreover, $\boldsymbol {g}_{sk}$ denotes a cause-specific vector-valued function of observed marker values. To model the baseline cause-specific hazard functions, we use B-splines, ie $h_{sk0} (t) = \exp \left\{ {\boldsymbol {B}_{sk}^{\mathrm{ \top }} (t)\boldsymbol {\psi }_{sk} } \right\}$. The interpretation of the parameters in terms of characterizing the missingness mechanism related to each event type as MCAR, MAR, and MNAR is similar: if both $\alpha _{sk1} = \alpha _{sk2} = 0$, the *k*th cause is either MCAR (if ${\boldsymbol {\phi }}_{sk} = \boldsymbol {\gamma }_{sk} = {\bf 0}$) or MAR (if ${\boldsymbol {\phi }}_{sk} \ne {\bf 0}$ or $\boldsymbol {\gamma }_{sk} \ne {\bf 0}$), whereas if $\alpha _{sk1} \ne 0$ or $\alpha _{sk2} \ne 0$, the *k*th failure cause is MNAR. Note that the cause-specific hazard functions, conditional on the random effects and previously observed marker values and visits, are assumed to be independent of current and future marker values. The parameters of the competing risk model are denoted by $\boldsymbol {\theta }_s^{\mathrm{ \top }} = (\boldsymbol {\theta }_{s1}^{\mathrm{ \top }} , \ldots , \boldsymbol {\theta }_{sK}^{\mathrm{ \top }} )$, where $\boldsymbol {\theta }_{sk}^{\mathrm{ \top }} = (\boldsymbol {\psi }_{sk}^{\mathrm{ \top }} , \boldsymbol {\gamma }_{sk}^{\mathrm{ \top }} , {\boldsymbol {\phi }}_{sk}^{\mathrm{ \top }} , \alpha _{sk1} , \alpha _{sk2} )$.

### 2.4 Estimation method

As described in the previous subsections, we have specified models, conditional on the random effects, for (i) the marginal distribution of *Y* (ie unconditionally on competing risk status), (ii) the conditional distribution of $N^* |Y$, and (iii) the cause-specific hazards conditional on *Y* and prior visits. This factorization consistently characterizes the joint evolution of the marker process, the visiting process, and the competing risk process. As clarified in Section S1, assuming noninformative right censoring, the entire likelihood for an individual is the product of the likelihood functions of the respective models. Thus, $f(\boldsymbol {Y}_i , N_i^{obs} , T_i , K_i ;\boldsymbol {\theta })$, suppressing conditioning on covariates for simplicity, where $N_i^{obs} = \left\{ {dN_i (t_{i1} ) = 1, \ldots , dN_i (t_{in_i } ) = 1} \right\}$ is a shorthand for the event “*n_i_* visits occurred at times $t_{i1} < t_{i2} < \ldots < t_{in_i }$”, can be factorized as

(2.4) $$f(\boldsymbol {Y}_i ;\boldsymbol {\theta }_l )\int f (N_i^{obs} |\boldsymbol {Y}_i , \boldsymbol {b}_i ;\boldsymbol {\theta }_v ) \times f(T_i , K_i |\boldsymbol {Y}_i , \boldsymbol {b}_i ;\boldsymbol {\theta }_s )f(\boldsymbol {b}_i |\boldsymbol {Y}_i ;\boldsymbol {\theta }_l )d\boldsymbol {b}_i ,$$

where $\boldsymbol {Y}_i \sim N(\boldsymbol {X}_i \boldsymbol {\beta }, \sigma ^2 \boldsymbol {I} + \boldsymbol {Z}_i \boldsymbol {DZ}_i^{\mathrm{ \top }} )$ and $\boldsymbol {b}_i |\boldsymbol {Y}_i \sim N(\boldsymbol {\mu }_i , \boldsymbol {C}_i )$, with $\boldsymbol {C}_i^{ - 1} = (\boldsymbol {D}^{ - 1} + \boldsymbol {Z}_i^{\mathrm{ \top }} \boldsymbol {Z}_i )$ and $\boldsymbol {\mu }_i = \boldsymbol {C}_i \boldsymbol {Z}_i^{\mathrm{ \top }} (\boldsymbol {Y}_i - \boldsymbol {X}_i \boldsymbol {\beta })/\sigma ^2$. The detailed form of the conditional likelihood for the competing risks, as implied by Equation (2.3), is

(2.5) $$\eqalign{ & f(T_i , K_i |{\boldsymbol {Y}}_i , {\boldsymbol {b}}_i ;{\boldsymbol {\theta }}_s ) = \prod\limits_{k = 1}^K ( \exp [{\boldsymbol {B}}_{sk}^{\mathrm{ \top }} (T_i ){\boldsymbol {\psi }}_{sk} + {\boldsymbol {\gamma }}_{sk}^{\mathrm{ \top }} \overline {\boldsymbol {X}} _{ski} (t_{in_i } ) + {\boldsymbol {\phi }}_{sk}^{\mathrm{ \top }} {\boldsymbol {g}}_{sk} \{ \bar Y_i (t_{in_i } )\} \cr & \quad + \alpha _{sk1} m_i (0) + \alpha _{sk2} m_i^\prime (T_i )])^{\delta _{ik} } \cr & \quad \times \exp ( - \sum\limits_{j = 1}^{n_i - 1} {\int_{t_{ij} }^{t_{ij + 1} } {\exp } } [{\boldsymbol {B}}_{sk}^{\mathrm{ \top }} (u){\boldsymbol {\psi }}_{sk} + {\boldsymbol {\gamma }}_{sk}^{\mathrm{ \top }} \overline {\boldsymbol {X}} _{ski} (t_{ij} ) \cr & \quad + {\boldsymbol {\phi }}_{sk}^{\mathrm{ \top }} {\boldsymbol {g}}_{sk} \{ \bar Y_i (t_{ij} )\} + \alpha _{sk1} m_i (0) + \alpha _{sk2} m_i^\prime (u)]du \cr & \quad - \int_{t_{in_i } }^{T_i } {\exp } [{\boldsymbol {B}}_{sk}^{\mathrm{ \top }} (u){\boldsymbol {\psi }}_{sk} + {\boldsymbol {\gamma }}_{sk}^{\mathrm{ \top }} \overline {\boldsymbol {X}} _{ski} (t_{in_i } ) \cr & \quad + {\boldsymbol {\phi }}_{sk}^{\mathrm{ \top }} \boldsymbol {g}_{sk} \{ \bar Y_i (t_{in_i } )\} + \alpha _{sk1} m_i (0) + \alpha _{sk2} m_i^\prime (u)]du). \cr}$$

The conditional likelihood of the visiting process, under the gap time model (2.1), is equal to

(2.6) $$\eqalign{ & f(N_i^{obs} |{\boldsymbol {Y}}_i , {\boldsymbol {b}}_i ;\boldsymbol {\theta }_v , {\boldsymbol {\beta }}) = \prod\limits_{j = 1}^{n_i - 1} {\exp } [{\boldsymbol {B}}_v^{\mathrm{ \top }} (u_{ij} ){\boldsymbol {\psi }}_v + {\boldsymbol {\gamma }}_v^{\mathrm{ \top }} \overline {\boldsymbol {X}} _{vi} (t_{ij} ) + {{\boldsymbol {\phi }}}_v^{\mathrm{ \top }} {\boldsymbol {g}}_v \{ \bar Y_i (t_{ij} )\} \cr & \quad + \alpha _{v1} m_i (0) + \alpha _{v2} m_i^\prime (t_{ij} + u_{ij} )] \cr & \quad \times \exp ( - \sum\limits_{j = 1}^{n_i } {\int_0^{u_{ij} } {\exp } } [{\boldsymbol {B}}_v^{\mathrm{ \top }} (s){\boldsymbol {\psi }}_v + {\boldsymbol {\gamma }}_v^{\mathrm{ \top }} \overline {\boldsymbol {X}} _{vi} (t_{ij} ) \cr & \quad + {\boldsymbol {\phi }}_v^{\mathrm{ \top }} \boldsymbol {g}_v \{ \bar Y_i (t_{ij} )\} + \alpha _{v1} m_i (0) + \alpha _{v2} m_i^\prime (t_{ij} + s)]ds). \cr}$$

When an intensity-based model (2.2) using calendar time is applied, the form of the conditional likelihood function is similar (Cook and Lawless 2007), ie

(2.7) $$\eqalign{ & \prod\limits_{j = 1}^{n_i - 1} {\exp } [{\boldsymbol {B}}_v^{\mathrm{ \top }} (t_{ij + 1} ){\boldsymbol {\psi }}_v + {\boldsymbol {\gamma }}_v^{\mathrm{ \top }} \overline {\boldsymbol {X}} _{vi} (t_{ij} ) + {\boldsymbol {\phi }}_v^{\mathrm{ \top }} {\boldsymbol {g}}_v \{ \bar Y_i (t_{ij} )\} + \alpha _{v1} m_i (0) + \alpha _{v2} m_i^\prime (t_{ij + 1} )] \cr & \qquad \times \exp ( - \sum\limits_{j = 1}^{n_i - 1} {\int_{t_{ij} }^{t_{ij + 1} } {\exp } } [{\boldsymbol {B}}_v^{\mathrm{ \top }} (s){\boldsymbol {\psi }}_v + {\boldsymbol {\gamma }}_v^{\mathrm{ \top }} \overline {\boldsymbol {X}} _{vi} (t_{ij} ) + {\boldsymbol {\phi }}_v^{\mathrm{ \top }} {\boldsymbol {g}}_v \{ \bar Y_i (t_{ij} )\} \cr & \qquad + \alpha _{v1} m_i (0) + \alpha _{v2} m_i^\prime (s)]ds - \int_{t_{in_i } }^{T_i } {\exp } [{\boldsymbol {B}}_v^{\mathrm{ \top }} (s){\boldsymbol {\psi }}_v + {\boldsymbol {\gamma }}_v^{\mathrm{ \top }} \overline {\boldsymbol {X}} _{vi} (t_{in_i } ) + {\boldsymbol {\phi }}_v^{\mathrm{ \top }} {\boldsymbol {g}}_v \{ \bar Y_i (t_{in_i } )\} \cr & \qquad + \alpha _{v1} m_i (0) + \alpha _{v2} m_i^\prime (s)]ds). \cr}$$

To approximate the integrals and the slope of the “true” marker values in the conditional likelihoods above, we applied the 15-point Gauss-Kronrod rule and the central difference approximation, respectively. To approximate the integral over the random effects, we propose using either Gauss-Hermite rules or Monte Carlo integration.

For the Gauss-Hermite method, note that after making the transformation $\boldsymbol {a}_i = 2^{ - 1/2} \boldsymbol {B}_i (\boldsymbol {b}_i - \boldsymbol {\mu }_i )$ in Equation (2.4), where $\boldsymbol {C}_i^{ - 1} = \boldsymbol {B}_i^{\mathrm{ \top }} \boldsymbol {B}_i , \;f(\boldsymbol {b}_i |\boldsymbol {Y}_i ;\boldsymbol {\theta }_l )$ essentially becomes the importance density. This is similar to the pseudo-adaptive rule (Rizopoulos 2012), but the posterior mode, given only the marker model, can be updated at each iteration. Then the second term of the marginal likelihood, ie $f(N_i^{obs} , T_i , K_i |\boldsymbol {Y}_i ;\boldsymbol {\theta })$, can be approximated by

$$\pi ^{ - q/2} \sum\limits_{i_1 }^{N_{GH} } \ldots \sum\limits_{i_q }^{N_{GH} } {\omega _{i_1 } } \ldots \omega _{i_q } f(N_i^{obs} , T_i , K_i |\boldsymbol {Y}_i , \boldsymbol {b}_i = \boldsymbol {\mu }_i + \sqrt 2 \boldsymbol {B}_i^{ - 1} (a_{i_1 } , \ldots , a_{i_q } )^{\mathrm{ \top }} ;\boldsymbol {\theta }),$$

where $a_1 , \ldots , a_{N_{GH} }$ and $\omega _1 , \ldots , \omega _{N_{GH} }$ denote the standard Gauss-Hermite points and weights, respectively. When the dimension of the random effects is large, Gauss-Hermite becomes infeasible. Using the same idea, we can approximate the integral using (quasi) Monte Carlo integration by drawing $\{ \boldsymbol {b}_i^{(j)} \} _{j = 1}^{N_{mc} }$ from the $N(\boldsymbol {\mu }_i , \boldsymbol {C}_i )$ distribution

$$N_{mc}^{ - 1} \sum\limits_{j = 1}^{N_{mc} } f (N_i^{obs} , T_i , K_i |\boldsymbol {Y}_i , \boldsymbol {b}_i^{(j)} ;\boldsymbol {\theta }).$$

To calculate the maximum likelihood estimates of the model, we applied the BFGS algorithm, requiring an analytic or approximated score vector. For the *i*th individual, the score vector equals $S_i (\boldsymbol {\theta }) = \int A (\boldsymbol {\theta }, \boldsymbol {b}_i )f(\boldsymbol {b}_i |\boldsymbol {Y}_i , N_i^{obs} , T_i , K_i ;\boldsymbol {\theta })d\boldsymbol {b}_i$ (Rizopoulos 2012), where

$$A(\boldsymbol {\theta }, \boldsymbol {b}_i ) = {{\partial \log \left\{ {f(\boldsymbol {Y}_i |\boldsymbol {b}_i ;\boldsymbol {\theta }_l )f(N_i^{obs} |\boldsymbol {Y}_i , \boldsymbol {b}_i ;\boldsymbol {\theta }_v )f(T_i , K_i |\boldsymbol {Y}_i , \boldsymbol {b}_i ;\boldsymbol {\theta }_s )f(\boldsymbol {b}_i |\boldsymbol {\theta }_l )} \right\}} \over {\partial \boldsymbol {\theta }}}$$

is equal to the complete data score vector (given $\boldsymbol {b}_i$). Rearranging terms, this becomes equal to

(2.8) $$S_i (\boldsymbol {\theta }) = \int A (\boldsymbol {\theta }, \boldsymbol {b}_i ){{f(N_i^{obs} , T_i , K_i |\boldsymbol {Y}_i , \boldsymbol {b}_i ;\boldsymbol {\theta })} \over {f(N_i^{obs} , T_i , K_i |\boldsymbol {Y}_i ;\boldsymbol {\theta })}}f(\boldsymbol {b}_i |\boldsymbol {Y}_i ;\boldsymbol {\theta })d\boldsymbol {b}_i .$$

Note that the second term in the integral is the ratio of the conditional to the marginal density. Thus, the score vector can be approximated using the same GH or Monte Carlo points as those used for approximating the observed likelihood. To speed up calculations, $A(\boldsymbol {\theta }, \boldsymbol {b}_i )$ was calculated analytically and the exact formulas are presented in Section S2. $\boldsymbol {D}$ was parametrized in terms of each matrix logarithm (Thomadakis et al. 2020). The above-mentioned approach was coded in *R* and the SEs were based on the inverse Hessian at convergence.

## 3 Simulation studies

### 3.1 Data generating mechanisms and fitted models

A detailed simulation study was carried out to assess the performance of the proposed methodology under certain scenarios. Longitudinal marker data were generated by an LMM of the form $Y_i (t) = (\beta _0 + b_{i0} ) + (\beta _1 + b_{i1} )\log (t + 1) + (\beta _2 + b_{i2} )(t/10)^3 + {\mathrm{ \epsilon }}_i (t)$, where $(b_{i0} , b_{i1} , b_{i2} )^{\mathrm{ \top }} \sim N({\bf 0}, \boldsymbol {D})$ and ${\mathrm{ \epsilon }}_i (t)\sim N(0, \sigma ^2 )$. To generate visit times, we assumed two scenarios for the visiting process, with a maximum study duration of 10 yrs. In the first scenario (scenario I), a proportional hazard model was adopted for the gap times between visits, ie $h_{vj} (u) = h_{v0} (u)\exp \left\{ {\phi _{v1} y_i (t_{ij} ) + \gamma _{v1} t_{ij} + \gamma _{v2} (u_{ij - 1} - 0.15)I(j > 1) + \alpha _{v1} m_i (0) + \alpha _{v2} m_i^\prime (t_{ij} + u)} \right\}, \;j = 1, 2, \ldots$, where $h_{v0} (u)$ denotes a common baseline hazard function, which is a function of time since the most recent visit, *u*. Since a previous gap time for the first visit time does not exist, to reduce an inconsistency in the interpretation of $h_{v0} (u)$ between the first and the remaining gap times, we centered $u_{ij - 1}$ at 0.15, which was the median first visit time in our motivating example. Thus, we assume that $h_{vj} (u) = h_{v1} (u)$ if $u_{ij - 1} = 0.15$ and $\gamma _{v1} = 0$, with all other covariates being equal. As a second scenario (scenario II), we generated visit times according to an intensity-based model using calendar time: $E\left\{ {dN_i^{\mathrm{ \star }} (t)} \right\} = \lambda _{v0} (t)\exp \{ \phi _{v1} y_i (t_{ij} ) + \gamma _{v1} (u_{ij - 1} - 0.15)I(j > 1) + \alpha _{v1} m_i (0) + \alpha _{v2} m_i^\prime (t)\}$, for $t_{ij} \le t < t_{ij + 1}$.

After simulating visit times and marker data, we generated competing risk data. Two proportional cause-specific hazard models: $h_1 (t) = h_{s10} (t)\exp \{ \phi _{s11} y_i (t_{ij} ) + \gamma _{s11} W_i + \alpha _{s11} m_i (0) + \alpha _{s12} m_i^\prime (t)\}$ and $h_2 (t) = h_{s20} (t)\exp \{ \phi _{s21} y_i (t_{ij} ) + \gamma _{s21} W_i + \gamma _{s22} (u_{ij - 1} - 0.15)I(j > 1) + \alpha _{s21} m_i (0) + \alpha _{s22} m_i^\prime (t)\}$, were assumed for cause 1 (death) and cause 2 (disengagement from care), respcectively, where $t_{ij} \le t < t_{ij + 1}$ and *W_i_* is a binary covariate. The true parameter values were based on our motivating example and are presented in Tables 1 and 2, with the shape of the baseline hazard functions described in Section S3. Visit times and competing risk times were generated by the inverse CDF theorem, utilizing the integrate and uniroot function in R to numerically solve the equations. Both scenarios led to approximately 11 visits per individual on average and 11.3% and 14.6% event rates for cause 1 and 2, respectively. data Six Hundred sets including 1,000 individuals were generated for each scenario.

**Table 1:** Simulation study results, where the data have been generated by the proposed model using a gap time visiting process (scenario I). Fitted model (i) is correctly specified (“Correctly specified”) and (ii) ignores the visiting process (“Ignoring visiting process”).

Parameter	True	Est	Bias	MSE	ASE	MCSD	Cov.	Est	Bias	MSE	ASE	MCSD	Cov.
**Longitudinal**	**Correctly specified**							**Ignoring visiting process**					
Intercept (*β*_0_)	17.20	17.190	-0.010	0.043	0.216	0.208	95.5	17.143	-0.057	0.047	0.217	0.208	94.8
$\log (t + 1)$ (*β*_1_)	4.83	4.844	0.014	0.015	0.122	0.121	95.3	4.991	0.161	0.043	0.129	0.131	76.5
$(t/10)^3 $ (*β*_2_)	-2.80	-2.828	-0.028	0.177	0.390	0.420	93.0	-2.048	0.752	0.732	0.379	0.407	49.3
Mean marker value at 10 yrs	25.98	25.979	-0.003	0.164	0.370	0.405	93.2	27.062	1.080	1.316	0.356	0.387	16.8
**Visiting process**													
Observed marker value ($\phi _{v1} $)	0.02	0.020	0.000	0.000	0.002	0.002	94.5						
*t_ij_* ($\gamma _{v1} $)	-0.02	-0.020	-0.000	0.000	0.006	0.006	95.5						
Previous gap time ($\gamma _{v2} $)	-1.50	-1.500	-0.000	0.001	0.038	0.038	94.7						
$m_i (0)$ ($\alpha _{v1} $)	0.02	0.020	-0.000	0.000	0.003	0.003	94.3						
$m_i^\prime (t)$ ($\alpha _{v2} $)	0.20	0.199	- 0.001	0.000	0.010	0.011	92.5						
**Dropout cause 1**													
Observed marker value ($\phi _{s11} $)	-0.20	-0.201	-0.001	0.001	0.030	0.031	94.2	-0.207	-0.007	0.001	0.030	0.032	93.7
Binary covariate ($\gamma _{s11} $)	0.50	0.515	0.015	0.038	0.196	0.195	95.2	0.515	0.015	0.038	0.197	0.195	95.3
$m_i (0)$ ($\alpha _{s11} $)	-0.05	-0.052	-0.002	0.002	0.040	0.041	94.3	-0.042	0.008	0.002	0.042	0.045	91.8
$m_i^\prime (t)$ ($\alpha _{s12} $)	-0.10	-0.096	0.004	0.029	0.168	0.171	96.2	-0.044	0.056	0.056	0.202	0.231	92.3
**Dropout cause 2**													
Observed marker value ($\phi _{s21} $)	-0.02	-0.020	0.000	0.001	0.023	0.024	93.7	-0.027	-0.007	0.001	0.023	0.025	92.2
Previous gap time ($\gamma _{s21} $)	1.40	1.414	0.014	0.009	0.097	0.095	95.2	1.410	0.010	0.009	0.097	0.096	95.3
Binary covariate ($\gamma _{s22} $)	0.50	0.510	0.010	0.029	0.172	0.171	94.8	0.510	0.010	0.030	0.172	0.172	94.7
$m_i (0)$ ($\alpha _{s21} $)	-0.05	-0.052	-0.002	0.001	0.030	0.031	94.2	-0.038	0.012	0.001	0.032	0.034	91.5
$m_i^\prime (t)$ ($\alpha _{s22} $)	-0.20	-0.198	0.002	0.029	0.169	0.169	95.0	-0.074	0.126	0.065	0.213	0.221	89.2

Results from 600 replications with each data set including 1000 individuals. The true marker evolution was based on a model of the form $Y_i (t) = (\beta _0 + b_{i0} ) + (\beta _1 + b_{i1} )\log (t + 1) + (\beta _2 + b_{i2} )(t/10)^3 + {\mathrm{ \epsilon }}_i (t)$. “True” denotes the true parameter values; “Est” the mean of the estimates over the 600 replications; “Bias” the mean bias of estimates; “MSE” the mean squared error; “ASE” the average SE, “MCSD” the empirical Monte carlo deviation of estimates and “Cov.” the empirical coverage probability (%) of the confidence intervals.

**Table 2:** Simulation study results, where the data have been generated by the proposed model using an intensity-based model (scenario II). Fitted model (i) is correctly specified (“Correctly specified”) and (ii) ignores the visiting process (“Ignoring visiting process”).

Parameter	True	Est	Bias	MSE	ASE	MCSD	Cov.	Est	Bias	MSE	ASE	MCSD	Cov.
**Longitudinal**													
Intercept (*β*_0_)	17.20	17.211	0.011	0.044	0.217	0.210	96.0	17.137	-0.063	0.048	0.217	0.210	94.7
$\log (t + 1)$ (*β*_1_)	4.83	4.827	-0.003	0.015	0.120	0.124	93.8	5.003	0.173	0.049	0.130	0.136	73.3
$(t/10)^3 $ (*β*_2_)	-2.80	-2.792	0.008	0.132	0.357	0.364	95.0	-2.345	0.455	0.344	0.356	0.371	73.7
Mean marker value at 10 yrs	25.98	25.994	0.012	0.109	0.333	0.330	94.5	26.790	0.808	0.765	0.332	0.334	31.5
**Visiting process**													
Observed marker value ($\phi _{v1} $)	0.02	0.020	0.000	0.000	0.002	0.002	94.8						
Previous gap time ($\gamma _{v1} $)	-1.00	-1.000	-0.000	0.001	0.022	0.023	94.7						
$m_i (0)$ ($\alpha _{v1} $)	0.02	0.020	-0.000	0.000	0.003	0.003	94.7						
$m_i^\prime (t)$ ($\alpha _{v2} $)	0.20	0.200	-0.000	0.000	0.011	0.012	95.2						
**Dropout cause 1**													
Observed marker value ($\phi _{s11} $)	-0.20	-0.201	-0.001	0.001	0.030	0.030	95.0	-0.212	-0.012	0.001	0.030	0.031	92.3
Binary covariate ($\gamma _{s11} $)	0.50	0.484	-0.016	0.037	0.193	0.190	94.3	0.484	-0.016	0.037	0.194	0.191	95.0
$m_i (0)$ ($\alpha _{s11} $)	-0.05	-0.053	-0.003	0.002	0.039	0.040	95.5	-0.030	0.020	0.002	0.042	0.044	89.5
$m_i^\prime (t)$ ($\alpha _{s12} $)	-0.10	-0.110	-0.010	0.028	0.159	0.166	95.0	0.023	0.123	0.071	0.206	0.236	88.5
**Dropout cause 2**													
Observed marker value ($\phi _{s21} $)	-0.02	-0.021	-0.001	0.000	0.022	0.021	94.5	-0.031	-0.011	0.001	0.023	0.022	93.2
Previous gap time ($\gamma _{s21} $)	1.40	1.408	0.008	0.009	0.094	0.094	94.7	1.413	0.013	0.009	0.095	0.096	94.0
Binary covariate ($\gamma _{s22} $)	0.50	0.505	0.005	0.030	0.169	0.172	94.5	0.504	0.004	0.030	0.170	0.173	95.0
$m_i (0)$ ($\alpha _{s21} $)	-0.05	-0.049	0.001	0.001	0.029	0.028	95.2	-0.028	0.022	0.002	0.031	0.032	89.0
$m_i^\prime (t)$ ($\alpha _{s22} $)	-0.20	-0.188	0.012	0.033	0.173	0.181	95.2	0.003	0.203	0.098	0.218	0.238	81.3

Results from 600 replications with each data set including 1000 individuals. The true marker evolution was based on a model of the form $Y_i (t) = (\beta _0 + b_{i0} ) + (\beta _1 + b_{i1} )\log (t + 1) + (\beta _2 + b_{i2} )(t/10)^3 + {\mathrm{ \epsilon }}_i (t)$. “True” denotes the true parameter values; “Est” the mean of the estimates over the 600 replications; “Bias” the mean bias of estimates; “MSE” the mean squared error; “ASE” the average SE, “MCSD” the empirical Monte carlo deviation of estimates and “Cov.” the empirical coverage probability (%) of the confidence intervals.

The marker and competing risk submodels were correctly specified in the fitted models. Under scenario I, we fitted the proposed model assuming that: (i) the model is correctly specified, (ii) the visiting process is ignored, (iii) the marker values are ignored in the visiting process (gap time), (iv) the intensity function is modeled as in scenario II, and (v) $N_i^{\mathrm{ \star }} (t^ - )$, through linear splines with a knot at 9, is added to the intensity-based joint model in (iv). Similarly, under scenario II, the following models were fitted assuming that (a) the model is correctly specified, (b) the visiting process is ignored, (c) the marker values are ignored in the visiting process (intensity-based), (d) gap time is used (linear on *t_ij_*), and (e) gap time is used with linear splines for *t_ij_* at 4 yrs. To assess model performance, we present the bias, the mean squared error (MSE), the Monte Carlo standard deviation, the average model-based standard error, and the empirical coverage probability of the confidence intervals. When the fitted visiting process is misspecified, we do not present results for the bias and coverage probabilities for the respective parameters.

### 3.2 Results when data have been generated by a gap time visiting process (scenario I)

The results from a correctly specified model and a corresponding model ignoring the visiting mechanism, when the data have been generated according to scenario I, are presented in Table 1. When correctly specified, the model yielded approximately unbiased estimates for all parameters, with coverage rates of confidence intervals being close to the nominal level (92.5%-96.2%). When the visiting model is ignored, though, biases arose for the fixed-effect parameters of the marker model, ie for *β*_1_ (True: 4.83, Mean estimate: 4.991) and *β*_2_ (True: -2.80, Mean estimate: -2.048). These estimates were also accompanied by poor coverage rates (76.5% and 49.3%, respectively). As expected, the estimated population-averaged marker value at 10 yrs was higher than the true one (True: 25.98, Mean estimate: 27.062), with very low coverage rate (16.8%). The coverage rates for most parameters of the competing risk models were close to 95%, but the estimates of the association parameters ($\alpha _{s11} , \;\alpha _{s12} , \;\alpha _{s21}$, and $\alpha _{s22}$) were underestimated (Table 1). The MSEs for the model ignoring the visiting process were higher than those from the correctly specified model, especially for the parameters of the marker model (Table 1).

The results for models (iii)–(v), under Scenario I, are presented in Tables S1 and S2. When marker values are ignored in the gap-time visiting process (model iii), small biases were observed, along with acceptable coverage rates, ranging from 92.3% to 96.2% (Table S1). Some evidence for bias in the estimated parameters associating latent marker slope with the competing risks was observed. However, these findings are under the assumption that the observed marker values have a minimal effect on visiting probabilities ($\phi _{v1} = 0.02$). For the intensity-based joint model (iv), significant biases were detected in the marker fixed-effect parameters, *β*_1_ and *β*_2_, with poor coverage rates of 70.6% and 69.3%, respectively (Table S2). Despite this, the bias in the estimated population-averaged marker value at 10 yrs was relatively small (True: 25.98, Mean estimate: 25.715), with a coverage rate of 87.3%. Incorporating $N_i^{\mathrm{ \star }} (t^ - )$ into the intensity-based model (model v) resulted in less biased estimates for *β*_1_ and *β*_2_, with improved coverage rates of 80.8% and 77.6%, respectively. Similarly, the estimates for the mean marker value at 10 yrs improved to some degree (True: 25.98, Mean estimate: 25.755, coverage rate 89.8%). The inclusion of $N_i^{\mathrm{ \star }} (t^ - )$ also reduced the mean squared error (MSE) for the marker model parameters. Most parameters of the competing risk model were estimated with small biases, except for the association parameters, which were underestimated by both models (Table S2).

### 3.3 Results when data have been generated by an intensity-based visiting process (scenario II)

The results from a correctly specified model and a corresponding model that ignores the visiting process, when data are simulated by an intensity-based visiting process, are shown in Table 2. Similar to the findings in subsection 3.2, the model performed satisfactorily for all parameters when correctly specified, exhibiting small biases and coverage rates between 93.8% and 96.0%. However, when the visiting process was ignored, the population-averaged marker value at 10 yrs was overestimated (True: 25.98, Mean estimate: 26.790, Coverage: 31.5%) and the association parameters for competing risks were underestimated (Table 2).

The results for models (c)–(e), under Scenario II, are presented in Tables S3 and S4. When marker values are ignored in the intensity-based visiting process, the estimated marker value at 10 yrs was slightly biased (True: 25.98, Mean estimate: 25.834), with coverage rate 92.8% (Table S3). The estimated parameter associating the latent marker slope with cause 1 was biased (True: -0.10, Mean estimate: -0.151), although the coverage rate was satisfactory (94.3%). Fitting a joint model with a gap-time visiting process (model d) led to moderately biased estimates for the marker fixed-effect parameters *β*_1_ and *β*_2_ (Table S4). However, the bias in the estimated population-averaged marker value at 10 yrs was relatively small (True: 25.98, Mean estimate: 26.157), with a coverage probability of 91.0%. As in subsection 3.2, improving the gap-time visiting process by including linear splines for *t_ij_* reduced the bias and the mean squared error (MSE), and improved the coverage rates for the parameters of the marker model (Table S4). For instance, for the population-averaged marker value at 10 yrs, the mean estimate was 25.988 (True: 25.98) with a coverage rate of 94.7%. Small biases were observed for the competing risk parameters (Table S4). Thus, the gap-time model fitted under an intensity-based visiting process performed better than the intensity-based model fitted under a gap-time model.

### 3.4 Additional simulation studies

To investigate the impact of ignoring marker values under a stronger association between the observed marker values and the visiting probabilities ($\phi _{v1} = 0.15$), we carried out an extra simulation study using a gap time visiting process. The model resulted in seriously biased estimated marker values at 10 yrs (True: 25.98, Mean estimate: 24.969), together with a low coverage rate of 53.8% (Table S5).

We also evaluated the performance of the proposed model under a smaller sample (*N* = 300). Similar to the findings in subsection 3.2, the model performed satisfactorily even with the smaller sample size *N* = 300, where the average numbers of events were 34.2 and 43.1 for causes 1 and 2, respectively. Ignoring the visiting process led to an overestimated marker value at 10 yrs, with a low coverage probability of 58.4% (Table S6).

### 3.5 Simulation studies including frailties in the visiting process

The performance of the proposed model when individual-specific frailties, $w_i \sim {\mathop{\rm Gamma}\nolimits} (1/\eta , 1/\eta )$, are incorporated into the proposed methodology was also evaluated.

#### 3.5.1 Gap-time visiting process

A similar proportional hazard model of the form $h_{vj} (u) = w_i h_{v0} (u)\exp \{ \phi _{v1} y_i (t_{ij} ) + \gamma _{v1} t_{ij} + \alpha _{v1} m_i (0) + \alpha _{v2} m_i^\prime (t_{ij} + u)\} , \;j = 1, 2, \ldots$, was assumed for the gap times. The remaining characteristics of the true data-generating mechanism were the same as those described in subsection 3.1. Four models were fitted assuming: (i) the model is correctly specified, (ii) the visiting process is ignored, (iii) a simple marginal model (without frailties) for the gap-time visiting process, and (iv) a more elaborate version of (iii) including the mean gap times of previous visits and $N_i^{\mathrm{ \star }} (t^ - )$ modeled via linear splines with a knot at 4 visits.

As expected, the model performed satisfactorily when correctly specified, yielding estimates with small biases and adequate coverage probabilities (Table S7). Consistently with previous findings, ignoring the visiting process led to overestimated mean marker values at 10 yrs and underestimated parameters associating latent marker slopes with competing events (Table S7). Fitting an oversimplified model for the visiting process without frailties (model iii) resulted in underestimated mean marker values at 10 yrs (True: 25.98, Mean estimate: 25.197) and a poor coverage probability of 33.9%, along with underestimated association parameters in the competing risk models (Table S8). Elaborating the model by including the mean gap time of previous visits and $N_i^{\mathrm{ \star }} (t^ - )$ (model iv) reduced bias in the marker values at 10 yrs (True: 25.98, Mean estimate: 25.711, Coverage: 85.9%) and alleviated biases in the competing risk models.

#### 3.5.2 Intensity-based visiting process using calendar time

A proportional intensity model using calendar time, $E\left\{ {dN_i^{\mathrm{ \star }} (t)} \right\} = w_i \lambda _{v0} (t)\exp \{ \phi _{v1} y_i (t_{ij} ) + \alpha _{v1} m_i (0) + \alpha _{v2} m_i^\prime (t)\}$, was applied to generate visit times. Four models, similar to those described in subsection 3.5.1 but adopting a calendar-time scale, were fitted.

The findings were largely consistent with those from subsubsection 3.5.1, ie ignoring the visiting process led to overestimated marker values and adopting an over-simplistic model without frailties resulted in seriously biased results. Fortunately, elaborating the model with information from previous visits improved performance to a large extent (Tables S9 and S10).

## 4 Application

We applied the proposed methodology to data from electronic health records of the Athens Multicenter AIDS Cohort Study (AMACS), a clinic-based cohort study of people with HIV. Our goal is to jointly model the CD4 count evolution since treatment initiation (ART), the visiting process, and competing risks (death or AIDS onset and disengagement from care). A corresponding joint model ignoring the visiting process was also fitted for comparison. Disengagement from care was defined, in collaboration with study physicians, as 1.5 yrs without any visit, with the time of disengagement defined as 0.75 yrs after the last visit time. We included data from individuals diagnosed after 1/1/2,000, aged 35 yrs or older, restricting to the two main risk groups, ie MSM (infected through sex between men) and PWID (people who inject drugs). *N* = 2, 315 individuals were included, of whom 83.0% and 17.0% were MSM and PWID, respectively, with 219 (9.5%; MSM: 8.6%, PWID: 13.5%) deaths or AIDS events and 340 (14.7%; MSM: 10.8%, PWID: 33.5%) disengagements from care. The mean time (SD) between visits was 5.4 (3.2) and 6.4 (4.9) mo for MSM and PWID, respectively, and the corresponding median (IQR) number of visits was 12 (6–20) and 6 (3–11), respectively. To model the CD4 count trends, we used an LMM with natural splines with 3 and 2 internal knots for the fixed and the random effects on the square root scale, respectively. The CD4 model allowed for group-specific slopes (MSM and PWID) and was adjusted for age at ART initiation, AIDS occurrence before ART, and year of HIV diagnosis. Baseline hazard functions for competing risks and visit probabilities were modeled through B-splines with 1 and 3 knots, respectively.

**Fig. 2:**
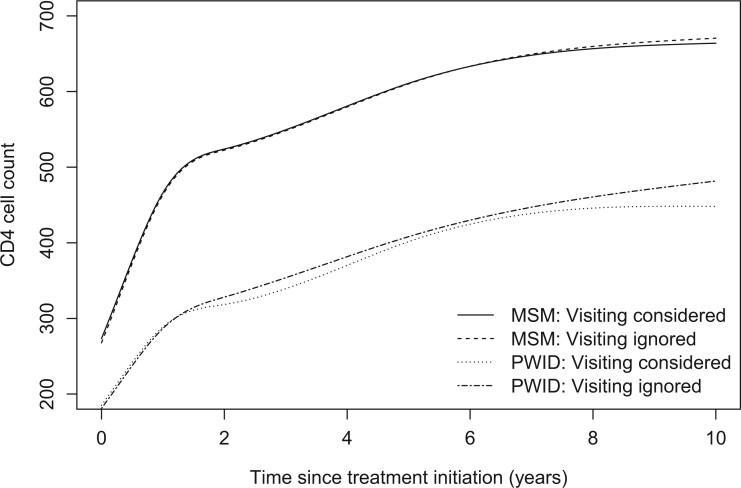
Estimated CD4 cell count evolution since treatment initiation based on the proposed approach that simultaneously models the visiting process through a gap-time approach including frailties and the two competing risks (death or aids onset and disengagement from care). A corresponding joint model ignoring the visiting process is also fitted. Estimates refer to individuals aged 35 yrs, without AIDS at treatment initiation, diagnosed on 1/1/2000. “MSM” denotes infected through sex between men and “PWID” denotes people who inject drugs.

The gap-time model provided a much better fit than the intensity-based model according to the AIC criterion, which was consistent with an informal explanatory analysis of the generalized residuals that suggested better performance for the gap-time model (Section S5). A corresponding gap-time model including frailties in the visiting process was further fitted, resulting in improved performance (LR-test=1,659). However, the remaining heterogeneity in the visiting model was moderate as indicated by the variance of the frailty, estimated to be 0.36 (95%CI: 0.32, 0.39). The following results refer to the gap-time frailty model. CD4 estimates by risk group are presented in Fig. 2. As expected from the medical literature, the estimated CD4 counts increased at a high rate up to about 1.5 yrs since ART initiation, followed by a much less steep slope thereafter. Ignoring the visiting process led to higher CD4 counts, which is consistent with our findings from the simulation study (Tables 1 and 2). However, the differences were much more pronounced for PWID compared to MSM. This could be attributed to the higher frequency of visits and the lower variability in the gap times among MSM compared to PWID. Detailed results for the CD4 model are presented in Table S11.

In the visiting and competing risk models, we also adjusted for known, possibly significant covariates, such as age at ART initiation, AIDS occurrence before ART, most recent HIV-RNA levels, and year of HIV diagnosis. The results are presented in Table 3. As for the visiting process there was a relatively small, although significant, effect of the observed CD4 counts on the visiting probabilities (Table 3). Interestingly, on top of the heterogeneity captured by frailties, there was a strong dependence on prior visits, since a smaller previous gap time is associated with higher visiting probabilities. Moreover, PWID had a 33% lower hazard of making a visit compared to MSM, adjusting for the remaining factors. The “true” CD4 value at baseline, $m_i (0)$, had only a small effect, although significant. The “true” CD4 rate of change on the square root scale, however, was more strongly associated with the frequency of visits: ie a one-unit increase in the latent CD4 slope was related to a 13% increase in the hazard of making a visit. Therefore, our model suggests that the visiting process is VNAR (informative), since it depends on latent CD4 counts, even after adjusting for visit history covariates and the observed most recent CD4 counts.

**Table 3:** Results from the proposed joint model fitted to data from the Athens Multicenter AIDS Cohort Study (AMACS).

Parameter	HR	95% CI	*P*
Visiting process			
Observed $\sqrt {{\rm CD4}} $ ($\sqrt {{\rm cells}/\mu L} $)	0.99	(0.98,0.99)	<0.001
*t_ij_* (yrs)	0.97	(0.95,0.98)	<0.001
Previous gap time (yrs)	0.64	(0.59,0.69)	<0.001
$I\{ N_i (t^ - ) > 1\} $	0.44	(0.41,0.47)	<0.001
$N_{i}(t^{-})I\{N_{i}(t^{-})>2\}$	0.98	(0.97,0.98)	<0.001
Age at ART initiation (yrs)	1	(1,1.01)	0.37
Prior AIDS	1.1	(0.99,1.22)	0.067
Undetectable HIV-RNA	0.74	(0.7,0.77)	<0.001
PWID/MSM	0.67	(0.61,0.73)	<0.001
Year of HIV diagnosis	0.91	(0.9,0.91)	<0.001
$m_i (0)$	1.01	(1,1.02)	0.013
$m_i^\prime (t)$	1.13	(1.12,1.15)	<0.001
Variance (*η*)	0.36	(0.32,0.39)	
Death/AIDS			
Observed $\sqrt {{\rm CD4}} $ (MSM) ($\sqrt {{\rm cells}/\mu L} $)	0.82	(0.79,0.85)	<0.001
Observed $\sqrt {{\rm CD4}} $ (PWID) ($\sqrt {{\rm cells}/\mu L} $)	0.92	(0.87,0.97)	0.001
Age at ART initiation (MSM) (yrs)	1.02	(1,1.04)	0.027
Age at ART initiation (PWID) (yrs)	1.07	(1.03,1.11)	0.001
PWID/MSM	0.03	(0,0.24)	0.001
Prior AIDS	0.91	(0.64,1.3)	0.612
Undetectable HIV-RNA	0.62	(0.43,0.89)	0.009
Year of HIV diagnosis	0.98	(0.95,1)	0.102
$m_i (0)$	1.02	(0.97,1.07)	0.357
$m_i^\prime (t)$	1	(0.87,1.16)	0.991
Disengagement			
Observed $\sqrt {{\rm CD4}} $ ($\sqrt {{\rm cells}/\mu L} $)	0.99	(0.96,1.02)	0.381
Previous gap time (yrs)	3.01	(2.38,3.8)	<0.001
$I\{ N_i (t^ - ) > 1\} $	0.74	(0.44,1.24)	0.256
Age at ART initiation (yrs)	1.01	(1,1.03)	0.042
Prior AIDS	1.1	(0.79,1.55)	0.574
Undetectable HIV-RNA	0.84	(0.62,1.15)	0.275
PWID/MSM	3.13	(2.39,4.09)	<0.001
Year of HIV diagnosis	1.01	(0.98,1.04)	0.616
$m_i (0)$	0.96	(0.93,1)	0.03
$m_i^\prime (t)$	0.82	(0.71,0.96)	0.011

Presented results refer to the parameters of a gap-time visiting process including a frailty term and the two competing risks (death or AIDS onset and disengagement from care). “HR” denotes the hazard ratio (estimate of the variance of frailties for *η*).

Results for the AIDS or death cause-specific hazard showed a strong dependence on the observed most recent CD4 counts, which makes the conditional independence assumption unreasonable. However, there was significant interaction with the risk group, with the hazard ratio (HR) being 0.82 in MSM and 0.92 in PWID. This is the case probably as PWID often die of causes related to drug use, which are independent of HIV progression. Similarly, interaction between risk group and age was also found. More importantly, no significant association with latent CD4 trajectories was suggested. Therefore, missingness due to death is estimated to be MAR, although this should be interpreted with caution due to the low number of events (9.5%).

Regarding the model for disengagement from care, a small insignificant effect of the observed most recent CD4 count was found (HR=0.99 per one square root increase). As expected by inspection of the descriptive statistics, PWID were three times as likely to disengage from care compared to MSM. Moreover, a 1-yr increase in the previous gap time was associated with a triple hazard for disengagement from care at current time. Finally, lower latent slope was significantly related to higher hazard for disengagement from care (HR=0.82 per one square/yr, *P* = 0.011), which implies that missingness due to disengagement from care is estimated to be MNAR, even upon adjusting for observed CD4 counts and prior visit times.

## 5 Discussion

In this article, we have proposed a unified approach to jointly model a normally distributed longitudinal marker, the visiting process, and time-to-event data in the form of competing risks. The visiting and cause-specific submodels condition on the observed marker history, prior visits, and marker random effects using latent baseline value and current slope. Our model fits within the shared-parameter framework, avoiding the assumption of conditional independence between the processes (Pullenayegum and Lim 2016). The visiting process in our approach can adopt either a gap time scale, through proportional hazard models, or a calendar time scale, through proportional intensity models, in contrast to most existing approaches assuming an intensity-based model with calendar time. Dependence between visit times of the same individual could be accounted for by conditioning on a frailty term and/or functions of previous visits. Simulation studies, when frailties are not included, affirm satisfactory model performance under correct specification. When the visiting process is ignored, though, serious biases arose for the marker model and the association parameters between the latent marker values and the competing risks. When a wrong time scale was adopted in the fitted visiting process, biases also occurred, with the gap time formulation being more robust than the intensity-based model (calendar time). In either case, elaborating the visiting process by prior visit history improved the performance. Similar findings were observed in the simulation studies including frailties. Adopting an over-simplistic model without frailties resulted in biased results, but, elaborating the model with information from previous visits improved performance to a large extent.

The proposed model was also fitted to data from the AMACS study, jointly modeling longitudinal CD4 data, the visiting process, and competing risks. The gap-time approach demonstrated notably superior fit, emphasizing the importance of not a priori assuming a calendar time scale. Our model revealed an informative visiting mechanism, as evidenced by a positive association between visiting probabilities and latent CD4 slope, even after conditioning on observed CD4 values, visit history, relevant covariates, and a frailty term. Thus, similar to our simulation findings, ignoring the visiting process led to overestimated CD4 values. It should be noted, however, that we used a “working” definition of disengagement and not the unknown true disengagement from care, which could be considered interval censored.

Our approach differs from existing ones in several ways. While many semiparametric models rely on the assumption that the marker model, visiting process, and time-to-event model are independent given shared or correlated random effects (Pullenayegum and Lim 2016), our model takes into account previously observed marker values and visit times. This alignment with the definitions of VNAR visiting processes and MNAR missing data mechanisms implies dependence on unobserved marker values after conditioning on the observed ones. This is an important advantage of the model since we showed that serious biases can occur if this assumption fails and the effect of the observed marker data is strong. Similar phenomena were seen when joint models of marker and time-to-event data are fitted under MAR dropout (Thomadakis et al. 2019).

Another important issue is the time scale upon which a model for the visiting process is built. Most approaches are intensity-based, assuming a calendar time, probably because their implementation resembles survival analysis methods using counting processes. However, our model can accommodate a gap time scale, which is also valid (Cook and Lawless 2007) and found to be superior in our real data analysis. In our simulation study, the fact that the gap time model was more robust than the intensity-based model (calendar time), when misspecified, is not surprising. It can be explained by the fact that the gap time model was able to approximately account for a calendar time trend by including *t_ij_* as a covariate, whereas the calendar time model did not take into account the time since the last visit.

In our simulation study, we conducted an indirect comparison with existing approaches by omitting observed marker values in the visiting process of the fitted model. Neuhaus et al. (2018) demonstrated that biases under informative visiting decrease with a higher proportion of scheduled visits, particularly affecting parameters associated with random effects (McCulloch et al. 2016). In our simulation study, all visits are irregular, and our focus was on parameters associated with marker evolution, which involve random effects. Thus, our findings do not fundamentally contradict those from previous studies. Another factor that could affect our simulation results is the ratio of random intercept variance to residual variance, which could be an important area for future exploration. Furthermore, as with all modeling approaches, enhancement of the model performance could be achieved by employing more flexible functional forms for the covariates. For instance, in our application, the relationship between clinical AIDS and CD4 may not be linear, at least for low CD4 counts. We intentionally refrained from using the current “true” marker value to sidestep potential identifiability issues. Given that we also adjust for the previously observed marker values, we hypothesized that discerning between two closely correlated key parameters would require an exceptionally large sample size or could potentially compromise the model’s robustness. Finally, the two time scales considered (gap/calendar time) could be combined (Cook and Lawless 2007), e.g. by utilizing two different baseline functions. However, this would lead to increased computational complexity.

To summarize, we have proposed a unified method to jointly model the evolution of a normally distributed longitudinal marker, the visiting process, and competing risks, relaxing the commonly used conditional independence assumption and adopting either a gap or calendar time scale. As questions concerning the informativeness of the visiting process in EHR data become more prevalent, our model stands as a valuable tool for addressing such inquiries.

## Supplementary Material

kxae041_Supplementary_Data

## Data Availability

Software in the form of R code is available at https://github.com/cthomadak/JointModelVisCompRisk.

## References

[kxae041-B1] Cook RJ , Lawless J. 2007. The statistical analysis of recurrent events. In: Gail M, Krickeberg K, Sarmet J, Tsiatis A, Wong W, editors. Statistics for biology and health. New York: Springer. p. 1–336.

[kxae041-B2] He X , Tong X, Sun J. 2009. Semiparametric analysis of panel count data with correlated observation and follow-up times. Lifetime Data Anal. 15:177–196. 19082711 10.1007/s10985-008-9105-1

[kxae041-B3] Huang C-Y , Wang M-C, Zhang Y. 2006. Analysing panel count data with informative observation times. Biometrika. 93:763–775. 23729818 10.1093/biomet/93.4.763 PMC3666563

[kxae041-B4] Liang Y , Lu W, Ying Z. 2009. Joint modeling and analysis of longitudinal data with informative observation times. Biometrics. 65:377–384. 18759841 10.1111/j.1541-0420.2008.01104.x

[kxae041-B5] Lin DY , Ying Z. 2001. Semiparametric and nonparametric regression analysis of longitudinal data. J Am Stat Assoc. 96:103–113.

[kxae041-B6] Lin DY , Ying Z. 2003. Semiparametric regression analysis of longitudinal data with informative drop-outs. Biostatistics. 4:385–398. 12925506 10.1093/biostatistics/4.3.385

[kxae041-B7] Lipsitz SR , Fitzmaurice GM, Ibrahim JG, Gelber R, Lipshultz S. 2002. Parameter estimation in longitudinal studies with outcome-dependent follow-up. Biometrics. 58:621–630. 12229997 10.1111/j.0006-341x.2002.00621.x

[kxae041-B8] Liu L , Huang X, O’Quigley J. 2008. Analysis of longitudinal data in the presence of informative observational times and a dependent terminal event, with application to medical cost data. Biometrics. 64:950–958. 18162110 10.1111/j.1541-0420.2007.00954.x

[kxae041-B9] McCulloch CE , Neuhaus JM, Olin RL. 2016. Biased and unbiased estimation in longitudinal studies with informative visit processes. Biometrics. 72:1315–1324. 26990830 10.1111/biom.12501 PMC5026863

[kxae041-B10] Molenberghs G , Beunckens C, Sotto C, Kenward MG. 2008. Every missingness not at random model has a missingness at random counterpart with equal fit. J R Stat Soc Ser B (Stat Methodol). 70:371–388.

[kxae041-B11] Neuhaus JM , McCulloch CE, Boylan RD. 2018. Analysis of longitudinal data from outcome-dependent visit processes: failure of proposed methods in realistic settings and potential improvements. Stat Med. 37:4457–4471. 30112825 10.1002/sim.7932

[kxae041-B12] Pullenayegum EM , Lim LSH. 2016. Longitudinal data subject to irregular observation: a review of methods with a focus on visit processes, assumptions, and study design. Stat Methods Med Res. 25:2992–3014. 24855119 10.1177/0962280214536537

[kxae041-B13] Rizopoulos D. 2012. Joint models for longitudinal and time-to-event data: with applications in R. Chapman & Hall/CRC Biostatistics Series. Boca Raton: CRC Press.

[kxae041-B14] Rubin DB. 1976. Inference and missing data. Biometrika. 63:581–592.

[kxae041-B15] Su W , Jiang H. 2018. Semiparametric analysis of longitudinal data with informative observation times and censoring times. J Appl Stat. 45:1978–1993.

[kxae041-B16] Sun J , Sun L, Liu D. 2007. Regression analysis of longitudinal data in the presence of informative observation and censoring times. J Am Stat Assoc. 102:1397–1406.

[kxae041-B17] Sun L , Song X, Zhou J, Liu L. 2012. Joint analysis of longitudinal data with informative observation times and a dependent terminal event. J Am Stat Assoc. 107:688–700.

[kxae041-B18] Thomadakis C , Meligkotsidou L, Pantazis N, Touloumi G. 2019. Longitudinal and time-to-drop-out joint models can lead to seriously biased estimates when the drop-out mechanism is at random. Biometrics. 75:58–68. 30357814 10.1111/biom.12986

[kxae041-B19] Thomadakis C , Meligkotsidou L, Pantazis N, Touloumi G. 2020. Misspecifying the covariance structure in a linear mixed model under mar drop-out. Stat Med. 39:3027–3041. 32452081 10.1002/sim.8589

[kxae041-B20] Troxel AB , Lipsitz SR, Harrington DP. 1998. Marginal models for the analysis of longitudinal measurements with nonignorable non-monotone missing data. Biometrika. 85:661–672.

[kxae041-B21] Yu G , Li Y, Zhu L, Zhao H, Sun J, Robison LL. 2019. An additive–multiplicative mean model for panel count data with dependent observation and dropout processes. Scand J Stat. 46:414–431. 10.1111/sjos.12357 PMC6586419 31223184

