# Supplementary material for: Shared parameter modeling of longitudinal data allowing for possibly informative visiting process and terminal event
Source: Biostatistics. 2024 Oct 23;26(1):kxae041. doi: 10.1093/biostatistics/kxae041 (PMC11911807; doi:10.1093/biostatistics/kxae041)
Supplement: kxae041_Supplementary_Data [file kxae041_supplementary_data.pdf]

Biostatistics (2024), **0**, 0, *pp.* 1–**??**
doi:10.1093/biostatistics/ThomadakisVisitingProcessAppendix

Web-based supporting materials for (Shared parameter modeling of longitudinal data allowing for possibly informative visiting process and terminal event) by Christos Thomadakis, Loukia Meligkotsidou, Nikos Pantazis, and Giota Touloumi

Christos Thomadakis*

Department of Hygiene, Epidemiology and Medical Statistics, Medical School, National and Kapodistrian University of Athens, Athens, Greece

cthomadak@med.uoa.gr

Loukia Meligkotsidou

Department of Mathematics, National and Kapodistrian University of Athens, Athens, Greece

Nikos Pantazis

Department of Hygiene, Epidemiology and Medical Statistics, Medical School, National and Kapodistrian University of Athens, Athens, Greece

Giota Touloumi

Department of Hygiene, Epidemiology and Medical Statistics, Medical School, National and Kapodistrian University of Athens, Athens, Greece

Summary

This document is the on-line supplementary material for the paper entitled "Shared parameter

*To whom correspondence should be addressed.

© The Author 2024. Published by Oxford University Press. All rights reserved. For permissions, please e-mail: journals.permissions@oup.com

modeling of longitudinal data allowing for possibly informative visiting process and terminal event". In Section S1, the likelihood function of the proposed model is outlined. Section S2 presents the analytic form of the complete data score vector of the model. In Section S2, we present the baseline hazard/intensity functions used in the true data generating process of the simulation study. Finally, in Sections S4 and S5, additional results are presented about the simulation study and the real application, respectively.

Key words: Joint modeling; Informative visiting; Linear mixed model; Observation process; Shared parameter models; Visiting process.

S1. Derivation of the likelihood function of the proposed model

In this section, we derive the form of the likelihood function for the proposed model based on the assumed models described in Equations (2.1), (2.2), and (2.3) of the main part of the manuscript. For simplicity in notation, we drop the subscript i, referring to individuals, and condition on parameter values. Similar to the main part of the manuscript, we assume that n visits have occurred at times $t_1 = 0 < t_2 < \ldots < t_n$ before the occurrence of the follow-up time $T = \min(T^{\star}, C)$, i.e., the minimum of the terminal event time, $T^{\star}$, and the non-informative censoring time, C. The aim of the following derivation is to ensure that the likelihood function, conditional on the random effects, is the product of three conventional likelihood functions for the three processes (marker, visiting, and competing risk), and that unobserved marker values (e.g., marker values between two consecutive visits) are not involved in the observed likelihood function.

Marker process

Similar to Ryu *and others* (2007), it is reasonable to characterize the marker process by the joint distribution of $Y(t)$ at a very large number of R discrete time points, $u_1 = 0 < u_2 < \ldots < u_R$, where $R \geqslant n$. We also assume that as $R \to \infty$, $u_R \to \infty$, and $t_j = u_i$, for some $i = 1, 2, \ldots, R$, for each $j = 1, 2, \ldots, n$, i.e. the sequence of time points $\{u_r\}_{r=1}^R$ is sufficiently large and includes the observed visit times. Thus, the marker process can be approximated by the joint distribution of $Y(t)$ at the time points $\{u_r\}_{r=1}^R$,

$$f\left[\{Y(t)\}_{t=0}^{\infty}|\boldsymbol{b}\right] \simeq \prod_{r=1}^{R} f\left\{Y(u_r)|\boldsymbol{b}\right\},$$

since $Y(t)|\boldsymbol{b}$ are independent for each time $t \geqslant 0$. Thus, for each possible partition of the time axis, $\{u_r\}_{r=1}^R$, the observed marker values are denoted by $\boldsymbol{Y}_O = \{Y(t_1), Y(t_2), \ldots, Y(t_n)\}^\top$ and the unobserved marker values by $\boldsymbol{Y}_M^{(R)} = \{Y(u_r) : r = 1, 2, \ldots, R, u_r \notin \{t_1, t_2, \ldots, t_n\}\}$. The complete marker data for each partition are denoted by $\boldsymbol{Y}^{(R)} = \begin{pmatrix} \boldsymbol{Y}_O \\ \boldsymbol{Y}_M^{(R)} \end{pmatrix}$.

Competing risk process

Recall that, based on Equation (2.3) of the main part of the manuscript, the cause-specific hazard functions are assumed to depend on past observed marker values and visits, conditional on the random effects, i.e. for $t_j \leqslant t < t_{j+1}$,

$$h_k\{t|N^\star(\cdot), Y(\cdot), \boldsymbol{b}\} = h_k\{t|\overline{\boldsymbol{X}}_{sk}(t_j), \overline{Y}(t_j), \boldsymbol{b}\},$$

which essentially means that the cause-specific hazard functions depend on the marker and visiting processes only through $\overline{Y}(t_j)$ and $\overline{\boldsymbol{X}}_{sk}(t_j)$, respectively. The notation $N^\star(\cdot)$ and $Y(\cdot)$ denotes all possible visits and observed marker values, respectively. Thus, the all-cause hazard for any terminal event, $h\{t|N^\star(\cdot), Y(\cdot), \boldsymbol{b}\} = \sum_{k=1}^K h_k\{t|\overline{\boldsymbol{X}}_{sk}(t_j), \overline{Y}(t_j), \boldsymbol{b}\}$, also depends only on previously observed marker values and visits. Ignoring contributions from non-informative censoring at C, it is implied that the likelihood function for the competing risks, conditional on the random

effects, the complete marker values and prior visits, is equal to

$$L_{CR} = \prod_{k=1}^{K} h_k\{T|N^\star(\cdot), Y(\cdot), \boldsymbol{b}\}^{\delta_k} \exp\left[-\sum_{k=1}^{K}\int_0^T h_k\{u|N^\star(\cdot), Y(\cdot), \boldsymbol{b}\}du\right],$$

which, by the assumptions of the model, becomes equal to

$$L_{CR} = \prod_{k=1}^{K} h_k\{T|\overline{\boldsymbol{X}}_{sk}(t_n), \overline{Y}(t_n), \boldsymbol{b}\}^{\delta_k} \exp\left[-\sum_{k=1}^{K}\sum_{j=1}^{n-1}\int_{t_j}^{t_{j+1}} h_k\{u|\overline{\boldsymbol{X}}_{sk}(t_j), \overline{Y}(t_j), \boldsymbol{b}\}du \right.$$
$$\left. -\sum_{k=1}^{K}\int_{t_n}^{T} h_k\{u|\overline{\boldsymbol{X}}_{sk}(t_n), \overline{Y}(t_n), \boldsymbol{b}\}du\right]. \tag{S1.1}$$

This, in turn, is equivalent to Equation (2.5) of the main part of the manuscript.

Visiting process

Since all characteristics of a counting process can be uniquely described through the intensity function and a gap-time model can be written in terms of the intensity function (e.g. page 40 Cook and Lawless, 2007), here we focus on the intensity function to describe the visiting process. In models (2.1) and (2.2) of the main part of the manuscript, the intensity function of the underlying visiting process, $N^\star(t)$, is assumed to depend on previously observed marker values and visit times, conditional on the random effects, i.e. for $t_j \leqslant t < t_{j+1}$

$$E\{dN^\star(t)|H(t), Y(\cdot), \boldsymbol{b}\} = E\left\{dN^\star(t)|\overline{\boldsymbol{X}}_v(t_j), \overline{Y}(t_j), \boldsymbol{b}\right\},$$

where $H(t) = \{N^\star(s) : 0 \leqslant s < t\}$ denotes the history of the visiting process at time t. This implies that the intensity function is assumed to depend only on previously observed marker values and visit times, through $\overline{Y}(t_j)$ and $\overline{\boldsymbol{X}}_v(t_j)$, respectively. However, the complete path of the underlying counting process, $N^\star(\cdot)$, is not observable due to the occurrence of the terminal event or right censoring at T. Recall also that the observable counting process for the visits is denoted by $N(t) = N^\star\{\min(t, T)\}$. Thus, to relate the intensity of the observable process, $E\{dN(t)\}$, to that of the underlying process, $E\{dN^\star(t)\}$, additional assumptions are generally required. According

to Cook and Lawless (2007, page 49), this becomes possible if $R(t) = I(T \geqslant t)$, the "at-risk" process, and $dN^{\star}(t)$ are conditionally independent given the history. In our model, the all-cause hazard for any terminal event is assumed to depend only on past visits and observed marker values (given $\boldsymbol{b}$) and the right-censoring time, C, is assumed to be non-informative, thus the conditional independence between $R(t)$ and $dN^{\star}(t)$, given the observed history and the random effects, is ensured. Therefore, as the following equation shows, the intensity of the observable process is equal to the intensity of the underlying process when an individual is "at risk" for making a visit

$$\begin{aligned} E\left\{dN(t)|H(t), Y(\cdot), R(t), \boldsymbol{b}\right\} &= E\left\{dN^{\star}(t)R(t)|H(t), Y(\cdot), R(t), \boldsymbol{b}\right\} \\ &= R(t)E\left\{dN^{\star}(t)|H(t), Y(\cdot), R(t), \boldsymbol{b}\right\} \\ &= R(t)E\left\{dN^{\star}(t)|\overline{\boldsymbol{X}}_v(t_j), \overline{Y}(t_j), \boldsymbol{b}\right\}, \end{aligned}$$

where $dN(t) = dN^{\star}(t)R(t)$ by definition, the third row follows from the conditional independence between $R(t)$ and $dN^{\star}(t)$ and the assumptions of the model. Thus, as discussed in Cook and Lawless (2007, pages 48-49), the conditional probability density for the outcome, "n visits occurring at times $t_1 < \ldots < t_n$", given the full marker values, is equal to

$$L_{VIS} = \prod_{j=1}^{n-1} E\left\{dN^{\star}(t_{j+1})|H(t_{j+1}), Y(\cdot), \boldsymbol{b}\right\} \exp\left[-\int_0^{\infty} R(u)E\left\{dN^{\star}(u)|H(u), Y(\cdot), \boldsymbol{b}\right\} du\right]$$

which, based on the assumptions of the model that the intensity function depends only on prior visits and previously observed marker values conditional on the random effects, becomes

$$\begin{aligned} L_{VIS} &= \prod_{j=1}^{n-1} E\left\{dN^{\star}(t_{j+1})|\overline{\boldsymbol{X}}_v(t_j), \overline{Y}(t_j), \boldsymbol{b}\right\} \exp\left[-\int_0^{T} E\left\{dN^{\star}(u)|H(u), Y(\cdot), \boldsymbol{b}\right\} du\right] \\ &= \prod_{j=1}^{n-1} E\left\{dN^{\star}(t_{j+1})|\overline{\boldsymbol{X}}_v(t_j), \overline{Y}(t_j), \boldsymbol{b}\right\} \exp\left[-\sum_{j=1}^{n-1}\int_{t_j}^{t_{j+1}} E\left\{dN^{\star}(u)|\overline{\boldsymbol{X}}_v(t_j), \overline{Y}(t_j), \boldsymbol{b}\right\} du \right. \\ &\quad \left. - \int_{t_n}^{T} E\left\{dN^{\star}(u)|\overline{\boldsymbol{X}}_v(t_n), \overline{Y}(t_n), \boldsymbol{b}\right\} du\right]. \end{aligned} \tag{S1.2}$$

It is, thus, verified that Equation S1.2 is equivalent to Equation (2.7) of the main part of the manuscript.

Complete likelihood conditional on the random effects

Using the standard multiplication rule of probability, the complete likelihood, conditional on the full marker data and the random effects, can be factorized as the marginal distribution of the marker values times the conditional likelihood of the visiting and competing risk processes given the full marker values. Thus, the complete likelihood, ignoring contributions of right-censoring due to C, equals

$$L_{comp} = f\left\{\boldsymbol{Y}^{(R)}|\boldsymbol{b}\right\} L_{VIS} L_{CR}.$$

It should be also noted that, for each possible partition of the time axis, $\{u_r\}_{r=1}^{R}$, the likelihood functions L_{VIS} and L_{CR} depend only on the observed marker data, $\boldsymbol{Y}_O$. The observed likelihood, conditional on the random effects, should include only the observed marker values, though. Since we have used a discrete time axis for the marker measurements and recalling that $Y(t)|\boldsymbol{b}$ are independent, the observed likelihood can be derived by integrating out the unobserved marker values, yielding

$$\begin{aligned} L_{obs} &= f\left(\boldsymbol{Y}_O|\boldsymbol{b}\right) \prod_{\substack{i=1,2\ldots,R \\ u_r \notin \{t_1,t_2,\ldots,t_n\}}} \int f\{Y(u_r)|\boldsymbol{b}\} dY(u_r) L_{VIS} L_{CR} \\ &= f\left(\boldsymbol{Y}_O|\boldsymbol{b}\right) L_{VIS} L_{CR}, \end{aligned} \tag{S1.3}$$

ensuring that only the observed marker values are involved in the likelihood function. Finally, Equation (2.4) follows by integrating the random effects out of $L_{obs} \times f(\boldsymbol{b})$, after dividing and multiplying by $f\left(\boldsymbol{Y}_O\right)$, which denotes the density of a multivariate Normal distribution.

S2. Analytic presentation of the observed score vector of the model

In the main part of the manuscript, we have shown that the observed vector of the ith individual is equal to

$$S_i(\boldsymbol{\theta}) = \int A(\boldsymbol{\theta}, \boldsymbol{b}_i) \frac{f(N_i^{obs}, T_i, K_i | \boldsymbol{Y}_i, \boldsymbol{b}_i; \boldsymbol{\theta})}{f(N_i^{obs}, T_i, K_i | \boldsymbol{Y}_i; \boldsymbol{\theta})} f(\boldsymbol{b}_i | \boldsymbol{Y}_i; \boldsymbol{\theta}) d\boldsymbol{b}_i. \tag{S2.4}$$

where the first term is the complete-data score vector and the second term is a ratio of the conditional to the marginal likelihood. As described in the main part of the manuscript, Equation (S2.4) can be approximated using either Gauss-Hermite quadrature or Monte Carlo integration. To complete this process, below we present the calculation of $A(\boldsymbol{\theta}, \boldsymbol{b}_i)$ analytically for the model with and without frailties.

S2.1 *Proposed model without frailties*

In this subsection, we present the calculation of $A(\boldsymbol{\theta}, \boldsymbol{b}_i)$ for models (2.1) and (2.2) of the main part of the manuscript, which do not include a frailty term. Since the likelihood function of the gap time visiting process, conditional on the random effects, is equal to

$$\begin{aligned} f(N_i^{obs} | \boldsymbol{Y}_i, \boldsymbol{b}_i; \boldsymbol{\theta}_v, \boldsymbol{\beta}) = \prod_{j=1}^{n_i - 1} \exp \Big[& \boldsymbol{B}_v^\top (u_{ij}) \boldsymbol{\psi}_v + \boldsymbol{\gamma}_v^\top \overline{\boldsymbol{X}}_{vi}(t_{ij}) + \boldsymbol{\phi}_v^\top \boldsymbol{g}_v \{ \overline{Y}_i(t_{ij}) \} \\ & + \alpha_{v1} m_i(0) + \alpha_{v2} m_i'(t_{ij} + u_{ij}) \Big] \times \exp \left\{ - \sum_{j=1}^{n_i} \int_0^{u_{ij}} \psi_{ij}(\boldsymbol{\theta}, \boldsymbol{b}_i) ds \right\}, \end{aligned} \tag{S2.5}$$

where $\psi_{ij}(\boldsymbol{\theta}, \boldsymbol{b}_i) = \exp \Big[\boldsymbol{B}_v^\top (s) \boldsymbol{\psi}_v + \boldsymbol{\gamma}_v^\top \overline{\boldsymbol{X}}_{vi}(t_{ij}) + \boldsymbol{\phi}_v^\top \boldsymbol{g}_v \{ \overline{Y}_i(t_{ij}) \} + \alpha_{v1} m_i(0) + \alpha_{v2} m_i'(t_{ij} + s) \Big]$, then

$$\frac{\partial \log f(N_i^{obs} | \boldsymbol{Y}_i, \boldsymbol{b}_i; \boldsymbol{\theta}_v, \boldsymbol{\beta})}{\partial \boldsymbol{\theta}_v} = \sum_{j=1}^{n_i - 1} \begin{bmatrix} \boldsymbol{B}_v(u_{ij}) \\ \overline{\boldsymbol{X}}_{vi}(t_{ij}) \\ \boldsymbol{g}_v \{ \overline{Y}_i(t_{ij}) \} \\ m_i(0) \\ m_i'(t_{ij} + u_{ij}) \end{bmatrix} - \sum_{j=1}^{n_i} \int_0^{u_{ij}} \begin{bmatrix} \boldsymbol{B}_v(s) \\ \overline{\boldsymbol{X}}_{vi}(t_{ij}) \\ \boldsymbol{g}_v \{ \overline{Y}_i(t_{ij}) \} \\ m_i(0) \\ m_i'(t_{ij} + s) \end{bmatrix} \psi_{ij}(\boldsymbol{\theta}, \boldsymbol{b}_i) ds.$$

A very similar formula can be obtained for an intensity-based visiting process using calendar time.

For the competing risk parameters, it follows that

$$\frac{\partial \log f(T_i, K_i|\boldsymbol{Y}_i, \boldsymbol{b}_i; \boldsymbol{\theta}_s)}{\partial \boldsymbol{\theta}_{sk}} = \delta_{ik} \begin{bmatrix} \boldsymbol{B}_{sk}^\top(T_i) \\ \overline{\boldsymbol{X}}_{ski}(t_{in_i}) \\ \boldsymbol{g}_{sk}\{\overline{Y}_i(t_{in_i})\} \\ m_i(0) \\ m_i'(T_i) \end{bmatrix}$$

$$- \sum_{j=1}^{n_i} \int_{t_{ij}}^{t_{ij+1}} \begin{bmatrix} \boldsymbol{B}_{sk}^\top(u) \\ \overline{\boldsymbol{X}}_{ski}(t_{ij}) \\ \boldsymbol{g}_{sk}\{\overline{Y}_i(t_{ij})\} \\ m_i(0) \\ m_i'(u) \end{bmatrix} \exp\Big[\boldsymbol{B}_{sk}^\top(u)\boldsymbol{\psi}_{sk} + \boldsymbol{\gamma}_{sk}^\top \overline{\boldsymbol{X}}_{ski}(t_{ij}) + \boldsymbol{\phi}_{sk}^\top \boldsymbol{g}_{sk}\{\overline{Y}_i(t_{ij})\}$$

$$+\alpha_{sk1} m_i(0) + \alpha_{sk2} m_i'(u)\Big] du$$

$$- \int_{t_{in_i}}^{T_i} \begin{bmatrix} \boldsymbol{B}_{sk}^\top(u) \\ \overline{\boldsymbol{X}}_{ski}(t_{in_i}) \\ \boldsymbol{g}_{sk}\{\overline{Y}_i(t_{in_i})\} \\ m_i(0) \\ m_i'(u) \end{bmatrix} \exp\Big[\boldsymbol{B}_{sk}^\top(u)\boldsymbol{\psi}_{sk} + \boldsymbol{\gamma}_{sk}^\top \overline{\boldsymbol{X}}_{ski}(t_{in_i}) + \boldsymbol{\phi}_{sk}^\top \boldsymbol{g}_{sk}\{\overline{Y}_i(t_{in_i})\}$$

$$+\alpha_{sk1} m_i(0) + \alpha_{sk2} m_i'(u)\Big] du.$$

The within-individual variance, σ^2, only appears in the conditional likelihood of the marker model, $f(\boldsymbol{Y}_i|\boldsymbol{b}_i;\boldsymbol{\theta})$. Letting $\kappa = \log(\sigma^2)$, it follows that the complete-data score vector for κ is equal to

$$\frac{\partial \log f(\boldsymbol{Y}_i|\boldsymbol{b}_i;\boldsymbol{\theta})}{\partial \kappa} = -\frac{n_i}{2} + \frac{1}{2e^\kappa}(\boldsymbol{Y}_i - \boldsymbol{X}_i\boldsymbol{\beta} - \boldsymbol{Z}_i\boldsymbol{b}_i)^\top(\boldsymbol{Y}_i - \boldsymbol{X}_i\boldsymbol{\beta} - \boldsymbol{Z}_i\boldsymbol{b}_i).$$

For the fixed effects, it easily follows that the complete-data score vector for the marker model is equal to

$$\frac{\partial \log f(\boldsymbol{Y}_i|\boldsymbol{b}_i;\boldsymbol{\theta})}{\partial \boldsymbol{\beta}} = \frac{1}{\sigma^2}\boldsymbol{X}_i^\top(\boldsymbol{Y}_i - \boldsymbol{X}_i\boldsymbol{\beta} - \boldsymbol{Z}_i\boldsymbol{b}_i).$$

Similarly, for the competing risk submodel,

$$\frac{\partial \log f(T_i, K_i|\boldsymbol{Y}_i, \boldsymbol{b}_i; \boldsymbol{\theta}_s)}{\partial \boldsymbol{\beta}} = \sum_{k=1}^{K} \delta_{ik} \left\{ \alpha_{sk1} \boldsymbol{X}_i(0) + \alpha_{sk2} \boldsymbol{X}_i'(T_i) \right\}$$

$$- \sum_{k=1}^{K} \sum_{j=1}^{n_i-1} \int_{t_{ij}}^{t_{ij+1}} \left\{ \alpha_{sk1} \boldsymbol{X}_i(0) + \alpha_{sk2} \boldsymbol{X}_i'(u) \right\} \exp \Big[\boldsymbol{B}_{sk}^\top(u) \boldsymbol{\psi}_{sk} + \boldsymbol{\gamma}_{sk}^\top \overline{\boldsymbol{X}}_{ski}(t_{ij}) + \boldsymbol{\phi}_{sk}^\top \boldsymbol{g}_{sk} \{ \overline{Y}_i(t_{ij}) \}$$

$$+ \alpha_{sk1} m_i(0) + \alpha_{sk2} m_i'(u) \Big] du$$

$$- \sum_{k=1}^{K} \int_{t_{in_i}}^{T_i} \left\{ \alpha_{sk1} \boldsymbol{X}_i(0) + \alpha_{sk2} \boldsymbol{X}_i'(u) \right\} \exp \Big[\boldsymbol{B}_{sk}^\top(u) \boldsymbol{\psi}_{sk} + \boldsymbol{\gamma}_{sk}^\top \overline{\boldsymbol{X}}_{ski}(t_{in_i}) + \boldsymbol{\phi}_{sk}^\top \boldsymbol{g}_{sk} \{ \overline{Y}_i(t_{in_i}) \}$$

$$+ \alpha_{sk1} m_i(0) + \alpha_{sk2} m_i'(u) \Big] du.$$

Finally, the complete-data score vector of the visiting process over the fixed effects, $\boldsymbol{\beta}$, is equal to

$$\frac{\partial \log f(N_i^{obs}|\boldsymbol{Y}_i, \boldsymbol{b}_i; \boldsymbol{\theta}_v, \boldsymbol{\beta})}{\partial \boldsymbol{\beta}} = \sum_{j=1}^{n_i-1} \left\{ \alpha_{v1} \boldsymbol{X}_i(0) + \alpha_{v2} \boldsymbol{X}_i'(t_{ij} + u_{ij}) \right\}$$

$$- \sum_{j=1}^{n_i} \int_0^{u_{ij}} \left\{ \alpha_{v1} \boldsymbol{X}_i(0) + \alpha_{v2} \boldsymbol{X}_i'(t_{ij} + s) \right\} \psi_{ij}(\boldsymbol{\theta}, \boldsymbol{b}_i) ds.$$

For the covariance matrix of the random effects, it follows that

$$\frac{\partial \log f(\boldsymbol{b}_i; \boldsymbol{\theta})}{\partial \operatorname{vec}(\boldsymbol{D})} = -\frac{1}{2} \operatorname{vec}(\boldsymbol{D}^{-1}) + \frac{1}{2} (\boldsymbol{D}^{-1} \otimes \boldsymbol{D}^{-1}) \operatorname{vec}(\boldsymbol{b}_i \boldsymbol{b}_i^\top).$$

Then, the derivative over $\operatorname{vec}\{\log(\boldsymbol{D})\}$ is calculated through the chain rule, where $\log(\boldsymbol{D})$ represents the matrix logarithm of $\boldsymbol{D}$, using the results provided in the supplementary material of Thomadakis *and others* (2020).

S2.2 *Proposed model with frailties*

The likelihood function of a gap-time visiting process for the ith individual, conditional on an individual-specific frailty, $w_i \sim \text{Gamma}(1/\eta, 1/\eta)$, is equal to

$$f(N_i^{obs}|\boldsymbol{Y}_i, \boldsymbol{b}_i, w_i; \boldsymbol{\theta}_v, \boldsymbol{\beta}) = w_i^{d_i} \prod_{j=1}^{n_i} \exp\Big[\boldsymbol{B}_v^\top(u_{ij})\boldsymbol{\psi}_v + \boldsymbol{\gamma}_v^\top \overline{\boldsymbol{X}}_{vi}(t_{ij}) + \boldsymbol{\phi}_v^\top \boldsymbol{g}_v\{\overline{Y}_i(t_{ij})\}$$
$$+\alpha_{v1} m_i(0) + \alpha_{v2} m_i'(t_{ij}+u_{ij})\Big]^{\delta_{vij}} \times \exp\left\{-w_i \sum_{j=1}^{n_i} \int_0^{u_{ij}} \psi_{ij}(\boldsymbol{\theta}, \boldsymbol{b}_i) ds\right\},$$

where δ_{vij} is an indicator of whether the jth gap time is censored or not: i.e. $\delta_{vij} = 1$, for $j = 1, 2, \ldots, n_i - 1$, and $\delta_{vij} = 1$, for $j = n_i$. Similarly, $d_i = \sum_{j=1}^{n_i} \delta_{vij} = n_i - 1$ is the number of observed gap times for the ith individual. As the prior distribution for the frailty is $w_i \sim \text{Gamma}(1/\eta, 1/\eta)$,

$$f(w_i; \eta) = \frac{w_i^{\frac{1}{\eta}-1} \exp\left(-\frac{1}{\eta} w_i\right)}{\eta^{\frac{1}{\eta}} \Gamma\left(\frac{1}{\eta}\right)}.$$

The marginal likelihood for the visiting process, i.e. the one obtained by integrating out the frailty term, is equal to

$$f(N_i^{obs}|\boldsymbol{Y}_i, \boldsymbol{b}_i; \boldsymbol{\theta}_v, \boldsymbol{\beta}) = \int_0^\infty f(N_i^{obs}|\boldsymbol{Y}_i, \boldsymbol{b}_i, w_i; \boldsymbol{\theta}_v, \boldsymbol{\beta}) f(w_i; \eta) dw_i.$$

By standard arguments, i.e. completing the density function of a Gamma random variable, the marginal likelihood, $f(N_i^{obs}|\boldsymbol{Y}_i, \boldsymbol{b}_i; \boldsymbol{\theta}_v, \boldsymbol{\beta})$, becomes equal to

$$\prod_{j=1}^{n_i} \exp\Big[\boldsymbol{B}_v^\top(u_{ij})\boldsymbol{\psi}_v + \boldsymbol{\gamma}_v^\top \overline{\boldsymbol{X}}_{vi}(t_{ij}) + \boldsymbol{\phi}_v^\top \boldsymbol{g}_v\{\overline{Y}_i(t_{ij})\} + \alpha_{v1} m_i(0) + \alpha_{v2} m_i'(t_{ij}+u_{ij})\Big]^{\delta_{vij}}$$
$$\times \; \frac{\eta^{d_i} \Gamma\left(d_i + \frac{1}{\eta}\right)}{\Gamma\left(\frac{1}{\eta}\right) \left\{1 + \eta \sum_{j=1}^{n_i} \int_0^{u_{ij}} \psi_{ij}(\boldsymbol{\theta}, \boldsymbol{b}_i) ds\right\}^{d_i + \frac{1}{\eta}}}.$$

Thus, the derivative of $\log\{f(N_i^{obs}|\boldsymbol{Y}_i, \boldsymbol{b}_i; \boldsymbol{\theta}_v, \boldsymbol{\beta})\}$ with respect to $(\boldsymbol{\psi}_v^\top, \boldsymbol{\gamma}_v^\top, \boldsymbol{\phi}_v^\top, \alpha_{v1}, \alpha_{v2})^\top$ is equal to

$$\sum_{j=1}^{n_i} \delta_{vij} \begin{bmatrix} \boldsymbol{B}_v(u_{ij}) \\ \overline{\boldsymbol{X}}_{vi}(t_{ij}) \\ \boldsymbol{g}_v\{\overline{Y}_i(t_{ij})\} \\ m_i(0) \\ m_i'(t_{ij}+u_{ij}) \end{bmatrix} - \left(d_i + \frac{1}{\eta}\right) \frac{\eta \sum_{j=1}^{n_i} \int_0^{u_{ij}} \begin{bmatrix} \boldsymbol{B}_v(s) \\ \overline{\boldsymbol{X}}_{vi}(t_{ij}) \\ \boldsymbol{g}_v\{\overline{Y}_i(t_{ij})\} \\ m_i(0) \\ m_i'(t_{ij}+s) \end{bmatrix} \psi_{ij}(\boldsymbol{\theta}, \boldsymbol{b}_i) ds}{1 + \eta \sum_{j=1}^{n_i} \int_0^{u_{ij}} \psi_{ij}(\boldsymbol{\theta}, \boldsymbol{b}_i) ds}.$$

The complete-data score vector of the visiting process with respect to the fixed effects, $\boldsymbol{\beta}$, is equal to

$$\frac{\partial \log f(N_i^{obs} | \boldsymbol{Y}_i, \boldsymbol{b}_i; \boldsymbol{\theta}_v, \boldsymbol{\beta})}{\partial \boldsymbol{\beta}} = \sum_{j=1}^{n_i} \delta_{vij} \left\{ \alpha_{v1} \boldsymbol{X}_i(0) + \alpha_{v2} \boldsymbol{X}_i'(t_{ij}+u_{ij}) \right\}$$
$$- \left(d_i + \frac{1}{\eta}\right) \frac{\eta \sum_{j=1}^{n_i} \int_0^{u_{ij}} \left\{ \alpha_{v1} \boldsymbol{X}_i(0) + \alpha_{v2} \boldsymbol{X}_i'(t_{ij}+s) \right\} \psi_{ij}(\boldsymbol{\theta}, \boldsymbol{b}_i) ds}{1 + \eta \sum_{j=1}^{n_i} \int_0^{u_{ij}} \psi_{ij}(\boldsymbol{\theta}, \boldsymbol{b}_i) ds}.$$

Finally, the complete-data score vector over η is equal to

$$\frac{\partial \log f(N_i^{obs} | \boldsymbol{Y}_i, \boldsymbol{b}_i; \boldsymbol{\theta}_v, \boldsymbol{\beta})}{\partial \eta} = \frac{d_i}{\eta} + \frac{\partial}{\partial \eta} \log \left\{ \frac{\Gamma\left(d_i + \frac{1}{\eta}\right)}{\Gamma\left(\frac{1}{\eta}\right)} \right\}$$
$$+ \frac{1}{\eta^2} \log \left\{ 1 + \eta \sum_{j=1}^{n_i} \int_0^{u_{ij}} \psi_{ij}(\boldsymbol{\theta}, \boldsymbol{b}_i) ds \right\} - \left(d_i + \frac{1}{\eta}\right) \frac{\sum_{j=1}^{n_i} \int_0^{u_{ij}} \psi_{ij}(\boldsymbol{\theta}, \boldsymbol{b}_i) ds}{1 + \eta \sum_{j=1}^{n_i} \int_0^{u_{ij}} \psi_{ij}(\boldsymbol{\theta}, \boldsymbol{b}_i) ds},$$

where, as shown in Duchateau and Janssen (2007, page 47),

$$\frac{\partial}{\partial \eta} \log \left\{ \frac{\Gamma\left(d_i + \frac{1}{\eta}\right)}{\Gamma\left(\frac{1}{\eta}\right)} \right\} = -I(d_i > 0) \sum_{l=0}^{d_i - 1} (\eta + l\eta^2)^{-1}.$$

Very similar formulas are obtained when an intensity-based model using calendar time is assumed. Specifically, the integration limits $\int_0^{u_{ij}} ds$ should be replaced by $\int_{t_{ij}}^{t_{ij+1}} ds$ and $\int_{t_{in_i}}^{T_i} ds$, if $j = 1, 2, \ldots, n_i - 1$ and $j = n_i$, respectively. Correspondingly, $t_{ij} + s$ in the integrand should be replaced by s.

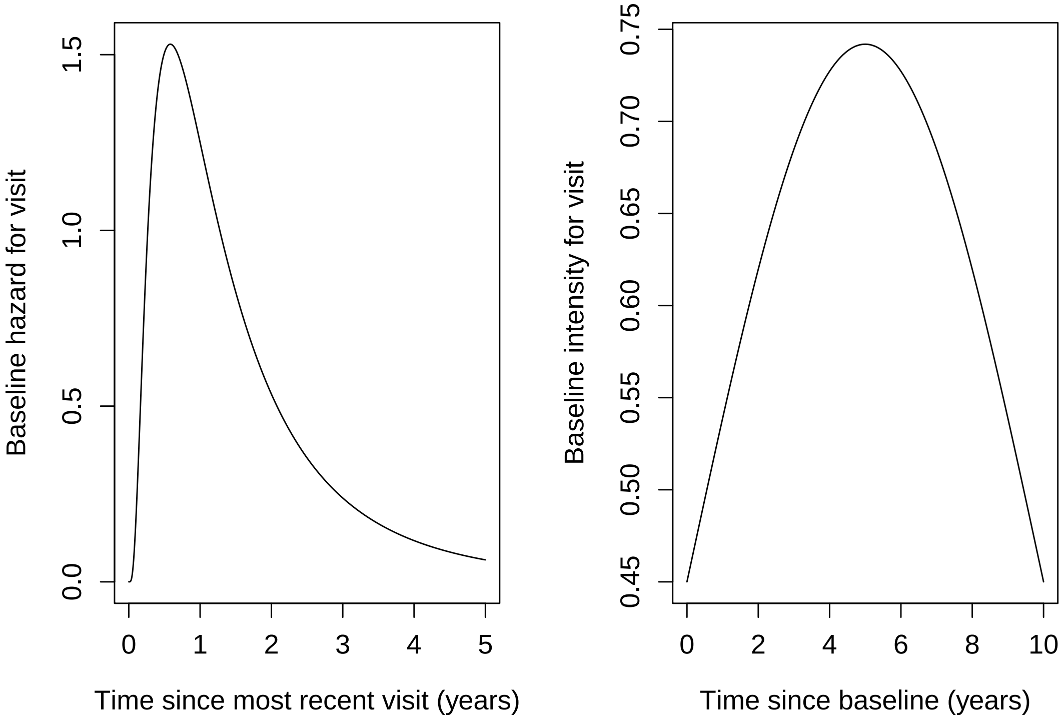

Fig. S1. Baseline functions of the visiting process used in the true data generating mechanism of the simulation study. The left panel shows the baseline hazard function of a gap time visiting process and the right panel shows the baseline intensity function of an intensity-based process using calendar time.

S3. BASELINE HAZARD FUNCTIONS IN THE DATA GENERATING PROCESS OF THE SIMULATION STUDY

Figures S1 and S2 present the true baseline hazard/intensity functions and baseline cause-specific hazard functions employed in the simulation studies described in the main part of the manuscript

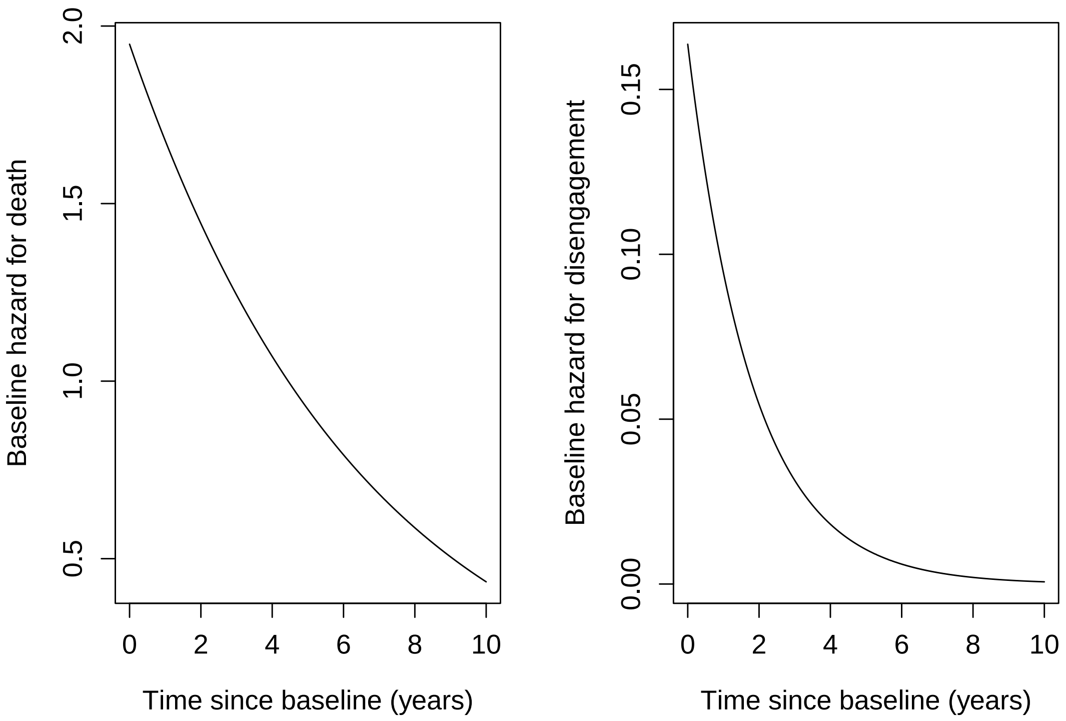

Fig. S2. Baseline cause-specific hazard functions used in the true data generating mechanism of the simulation study. The left panel shows the baseline cause-specific hazard for death and the right panel shows the baseline cause-specific hazard for disengagement from care.

S4. Simulation study results

S4.1 *Information on variance component parameters*

For all simulation scenarios presented in the main part of the manuscript, the variance-covariance matrix of the random effects is equal to

$$\boldsymbol{D} = \begin{pmatrix} 43.20 & -7.56 & -2.69 \\ -7.56 & 7.68 & -7.38 \\ -2.69 & -7.38 & 31.90 \end{pmatrix}$$

with the corresponding correlations being -0.42, -0.07, and -0.47. The within-individual standard deviation is equal to 2.81.

S4.2 *Scenario I*

Tables S1 and S2 present additional results from the simulation studies described in Section 3.2 of the main part of the manuscript.

Table S1. Simulation study results, where the data have been generated by the proposed model using a gap time visiting process (scenario I). The fitted joint model uses a gap time visiting process ignoring the observed marker values.

Parameter	True	Est	Bias	MSE	ASE	MCSD	Cov.
**Longitudinal**							
Intercept (β_0)	17.20	17.190	-0.010	0.043	0.215	0.208	95.3
$\log(t+1)$ (β_1)	4.83	4.798	-0.032	0.015	0.119	0.120	94.0
$(t/10)^3$ (β_2)	-2.80	-2.812	-0.012	0.182	0.391	0.426	92.3
Mean marker value at 10 years	25.98	25.883	-0.098	0.181	0.379	0.414	93.2
**Visiting process**							
Observed marker value (ϕ_{v1})	0.02						
t_{ij} (γ_{v1})	-0.02	0.006			0.006	0.006	
Previous gap time (γ_{v2})	-1.50	-1.492			0.038	0.038	
$m_i(0)$ (α_{v1})	0.02	0.036			0.002	0.002	
$m_i'(t)$ (α_{v2})	0.20	0.204			0.010	0.011	
**Dropout cause 1**							
Observed marker value (ϕ_{s11})	-0.20	-0.196	0.004	0.001	0.030	0.031	93.8
Binary covariate (γ_{s11})	0.50	0.516	0.016	0.038	0.196	0.195	95.3
$m_i(0)$ (α_{s11})	-0.05	-0.062	-0.012	0.002	0.040	0.041	93.5
$m_i'(t)$ (α_{s12})	-0.10	-0.149	-0.049	0.031	0.171	0.169	96.2
**Dropout cause 2**							
Observed marker value (ϕ_{s21})	-0.02	-0.016	0.004	0.001	0.024	0.025	93.8
Previous gap time (γ_{s21})	1.40	1.413	0.013	0.009	0.098	0.095	95.2
Binary covariate (γ_{s22})	0.50	0.511	0.011	0.030	0.172	0.171	94.8
$m_i(0)$ (α_{s21})	-0.05	-0.058	-0.008	0.001	0.030	0.031	94.0
$m_i'(t)$ (α_{s22})	-0.20	-0.249	-0.049	0.033	0.177	0.175	94.7

Results from 600 replications with each data set including 1000 individuals. The true marker evolution was based on a model of the form $Y_i(t) = (\beta_0 + b_{i0}) + (\beta_1 + b_{i1})\log(t+1) + (\beta_2 + b_{i2})(t/10)^3 + \epsilon_i(t)$. "True" denotes the true parameter values; "Est" the mean of the estimates over the 600 replications; "Bias" the mean bias of estimates; "MSE" the mean squared error; "ASE" the average SE, "MCSD" the empirical Monte carlo deviation of estimates and "Cov." the empirical coverage probability (%) of the confidence intervals.

Table S2. Simulation study results, where the data have been generated by the proposed model under a gap time visiting process (scenario I). Fitted models assume that the intensity function is modelled as in scenario II ("Intensity model"), and $N_i(t^-)$, through linear splines with a knot at 9, is added to the intensity-based joint model ("Intensity model + $N_i(t^-)$").

Parameter	True	Est	Bias	MSE	ASE	MCSD	Cov.	Est	Bias	MSE	ASE	MCSD	Cov.
**Longitudinal**		**Intensity model**						**Intensity model + $N_i(t^-)$**					
Intercept (β_0)	17.20	17.123	-0.077	0.049	0.218	0.208	94.0	17.138	-0.062	0.047	0.217	0.208	94.3
$\log(t+1)$ (β_1)	4.83	5.012	0.182	0.048	0.124	0.123	70.6	4.971	0.141	0.035	0.124	0.123	80.8
$(t/10)^3$ (β_2)	-2.80	-3.425	-0.625	0.651	0.446	0.510	69.3	-3.303	-0.503	0.502	0.440	0.499	77.6
Mean marker value at 10 years	25.98	25.715	-0.267	0.315	0.433	0.494	87.3	25.755	-0.227	0.280	0.428	0.478	89.8
**Visiting process**													
Observed marker value (ϕ_{v1})		0.011			0.002	0.003		0.012			0.003	0.002	
Previous gap time (γ_{v1})		-1.602			0.037	0.043		-1.612			0.041	0.052	
$N_i(t^-)$ (slope up to 9) (γ_{v2})								-0.079			0.008	0.016	
$N_i(t^-)$ (after 9) (γ_{v3})								0.035			0.005	0.005	
$m_i(0)$ (α_{v1})		0.018			0.003	0.003		0.017			0.003	0.003	
$m_i'(t)$ (α_{v2})		0.115			0.012	0.011		0.128			0.013	0.012	
**Dropout cause 1**													
Observed marker value (ϕ_{s11})	-0.20	-0.208	-0.008	0.001	0.030	0.032	93.8	-0.207	-0.007	0.001	0.030	0.032	93.5
Binary covariate (γ_{s11})	0.50	0.514	0.014	0.038	0.196	0.194	95.5	0.515	0.015	0.038	0.196	0.195	95.3
$m_i(0)$ (α_{s11})	-0.05	-0.041	0.009	0.002	0.041	0.043	93.0	-0.043	0.007	0.002	0.041	0.043	93.0
$m_i'(t)$ (α_{s12})	-0.10	-0.062	0.038	0.043	0.190	0.203	95.5	-0.063	0.037	0.042	0.187	0.201	95.7
**Dropout cause 2**													
Observed marker value (ϕ_{s21})	-0.02	-0.025	-0.005	0.001	0.024	0.025	93.3	-0.024	-0.004	0.001	0.024	0.025	93.2
Previous gap time (γ_{s21})	1.40	1.408	0.008	0.009	0.097	0.096	95.0	1.408	0.008	0.009	0.097	0.096	95.0
Binary covariate (γ_{s22})	0.50	0.510	0.010	0.030	0.172	0.172	94.7	0.511	0.011	0.030	0.172	0.172	94.7
$m_i(0)$ (α_{s21})	-0.05	-0.043	0.007	0.001	0.031	0.032	93.0	-0.044	0.006	0.001	0.031	0.032	93.7
$m_i'(t)$ (α_{s22})	-0.20	-0.131	0.069	0.040	0.193	0.189	93.3	-0.139	0.061	0.038	0.191	0.185	94.8

Results from 600 replications with each data set including 1000 individuals. The true marker evolution was based on a model of the form $Y_i(t) = (\beta_0 + b_{i0}) + (\beta_1 + b_{i1}) \log(t+1) + (\beta_2 + b_{i2})(t/10)^3 + \epsilon_i(t)$. "True" denotes the true parameter values; "Est" the mean of the estimates over the 600 replications; "Bias" the mean bias of estimates; "MSE" the mean squared error; "ASE" the average SE, "MCSD" the empirical Monte carlo deviation of estimates and "Cov." the empirical coverage probability (%) of the confidence intervals.

S4.3 *Scenario II*

Tables S3 and S4 present additional results from the simulation studies described in Section 3.3 of the main part of the manuscript.

Table S3. Simulation study results, where the data have been generated by the proposed model using an intensity-based visiting process (scenario II). The fitted joint model uses a intensity-based visiting process ignoring the observed marker values.

Parameter	True	Est	Bias	MSE	ASE	MCSD	Coverage
**Longitudinal**							
Intercept (β_0)	17.20	17.182	-0.018	0.045	0.216	0.210	95.7
$\log(t+1)$ (β_1)	4.83	4.820	-0.010	0.015	0.116	0.122	93.0
$(t/10)^3$ (β_2)	-2.80	-2.905	-0.105	0.143	0.356	0.364	93.7
Mean marker value at 10 years	25.98	25.834	-0.148	0.133	0.340	0.334	92.8
**Visiting process**							
Observed marker value (ϕ_{v1})	0.02						
Previous gap time (γ_{v1})	-1.00	-1.013			0.022	0.024	
$m_i(0)$ (α_{v1})	0.02	0.038			0.002	0.002	
$m_i'(t)$ (α_{v2})	0.20	0.228			0.012	0.012	
**Dropout cause 1**							
Observed marker value (ϕ_{s11})	-0.20	-0.197	0.003	0.001	0.030	0.031	94.8
Binary covariate (γ_{s11})	0.50	0.485	-0.015	0.037	0.193	0.191	94.3
$m_i(0)$ (α_{s11})	-0.05	-0.061	-0.011	0.002	0.040	0.040	94.0
$m_i'(t)$ (α_{s12})	-0.10	-0.151	-0.051	0.030	0.163	0.165	94.3
**Dropout cause 2**							
Observed marker value (ϕ_{s21})	-0.02	-0.018	0.002	0.000	0.023	0.022	95.2
Previous gap time (γ_{s21})	1.40	1.405	0.005	0.009	0.094	0.094	94.5
Binary covariate (γ_{s22})	0.50	0.505	0.005	0.030	0.170	0.172	94.5
$m_i(0)$ (α_{s21})	-0.05	-0.053	-0.003	0.001	0.030	0.029	95.3
$m_i'(t)$ (α_{s22})	-0.20	-0.216	-0.016	0.036	0.182	0.189	95.2

Results from 600 replications with each data set including 1000 individuals. The true marker evolution was based on a model of the form $Y_i(t) = (\beta_0 + b_{i0}) + (\beta_1 + b_{i1})\log(t+1) + (\beta_2 + b_{i2})(t/10)^3 + \epsilon_i(t)$. "True" denotes the true parameter values; "Est" the mean of the estimates over the 600 replications; "Bias" the mean bias of estimates; "MSE" the mean squared error; "ASE" the average SE, "MCSD" the empirical Monte carlo deviation of estimates and "Cov." the empirical coverage probability (%) of the confidence intervals.

Table S4. Simulation study results, where the data have been generated by the proposed model using an intensity-based visiting process (scenario II). Fitted models use a gap time approach as in scenario I ("Gap time (t_{ij})") and a gap time approach including linear splines for t_{ij} ("Gap time (linear splines for t_{ij})") .

Parameter	True	Est	Bias	MSE	ASE	MCSD	Cov.	Est	Bias	MSE	ASE	MCSD	Cov.
**Longitudinal**		**Gap time (t_{ij})**						**Gap time (linear splines for t_{ij})**					
Intercept (β_0)	17.20	17.239	0.039	0.046	0.218	0.210	95.8	17.217	0.017	0.044	0.217	0.210	95.8
$\log(t+1)$ (β_1)	4.83	4.787	-0.043	0.018	0.126	0.129	93.7	4.817	-0.013	0.016	0.122	0.125	94.7
$(t/10)^3$ (β_2)	-2.80	-2.560	0.240	0.194	0.361	0.369	89.1	-2.779	0.021	0.137	0.362	0.369	95.2
Mean marker value at 10 years	25.98	26.157	0.175	0.141	0.331	0.331	91.0	25.988	0.006	0.112	0.336	0.334	94.7
**Visiting process**													
Observed marker value (ϕ_{v1})		0.029			0.002	0.002		0.021			0.002	0.003	
Previous gap time (γ_{v1})		-0.987			0.023	0.024		-1.013			0.023	0.024	
t_{ij} (γ_{v2})		-0.011			0.006	0.006							
t_{ij} (slope<4) (γ_{v3})								0.092			0.012	0.012	
t_{ij} (slope⩾4) (γ_{v4})								-0.079			0.009	0.010	
$m_i(0)$ (α_{v1})		0.010			0.003	0.003		0.018			0.003	0.003	
$m_i'(t)$ (α_{v2})		0.142			0.009	0.009		0.177			0.010	0.010	
**Dropout cause 1**													
Observed marker value (ϕ_{s11})	-0.20	-0.203	-0.003	0.001	0.030	0.030	95.2	-0.202	-0.002	0.001	0.030	0.030	95.0
Binary covariate (γ_{s11})	0.50	0.485	-0.015	0.037	0.193	0.191	94.5	0.484	-0.016	0.037	0.193	0.191	94.5
$m_i(0)$ (α_{s11})	-0.05	-0.052	-0.002	0.002	0.040	0.041	94.8	-0.054	-0.004	0.002	0.040	0.040	95.3
$m_i'(t)$ (α_{s12})	-0.10	-0.114	-0.014	0.031	0.165	0.174	93.8	-0.124	-0.024	0.029	0.161	0.168	94.2
**Dropout cause 2**													
Observed marker value (ϕ_{s21})	-0.02	-0.021	-0.001	0.000	0.022	0.021	94.3	-0.021	-0.001	0.000	0.022	0.021	94.7
Previous gap time (γ_{s21})	1.40	1.409	0.009	0.009	0.094	0.094	94.8	1.409	0.009	0.009	0.094	0.094	94.7
Binary covariate (γ_{s22})	0.50	0.506	0.006	0.030	0.170	0.173	94.5	0.505	0.005	0.030	0.170	0.173	94.5
$m_i(0)$ (α_{s21})	-0.05	-0.050	-0.000	0.001	0.028	0.028	95.3	-0.050	0.000	0.001	0.028	0.028	95.5
$m_i'(t)$ (α_{s22})	-0.20	-0.220	-0.020	0.036	0.175	0.188	94.0	-0.205	-0.005	0.034	0.173	0.183	95.2

Results from 600 replications with each data set including 1000 individuals. The true marker evolution was based on a model of the form $Y_i(t) = (\beta_0 + b_{i0}) + (\beta_1 + b_{i1})\log(t+1) + (\beta_2 + b_{i2})(t/10)^3 + \epsilon_i(t)$. "True" denotes the true parameter values; "Est" the mean of the estimates over the 600 replications; "Bias" the mean bias of estimates; "MSE" the mean squared error; "ASE" the average SE, "MCSD" the empirical Monte carlo deviation of estimates and "Cov." the empirical coverage probability (%) of the confidence intervals.

S4.4 *Additional results*

Tables S5 and S6 present additional results from the simulation studies described in Section 3.4 of the main part of the manuscript.

Table S5. Simulation study results, where the data have been generated by the proposed model using a gap time visiting process under a stronger correlation between the visiting probabilities and the observed marker values. The fitted joint model uses a gap time visiting process ignoring the observed marker values.

Parameter	True	Est	Bias	MSE	ASE	MCSD	Coverage
**Longitudinal**							
Intercept (β_0)	17.20	17.233	0.033	0.053	0.208	0.228	91.7
$\log(t+1)$ (β_1)	4.83	4.256	-0.574	0.346	0.097	0.129	0.0
$(t/10)^3$ (β_2)	-2.80	-2.469	0.331	0.365	0.531	0.505	90.7
Mean marker value at 10 years	25.98	24.969	-1.012	1.286	0.529	0.511	53.8
**Visiting process**							
Observed marker value (ϕ_{v1})	0.15						
t_{ij} (γ_{v1})	-0.02	0.122			0.005	0.009	
Previous gap time (γ_{v2})	-1.50	-1.276			0.042	0.045	
$m_i(0)$ (α_{v1})	0.02	0.152			0.003	0.005	
$m_i'(t)$ (α_{v2})	0.20	0.259			0.012	0.022	
**Dropout cause 1**							
Observed marker value (ϕ_{s11})	-0.20	-0.172	0.028	0.002	0.037	0.036	87.5
Binary covariate (γ_{s11})	0.50	0.506	0.006	0.025	0.168	0.159	95.8
$m_i(0)$ (α_{s11})	-0.05	-0.086	-0.036	0.004	0.051	0.050	91.4
$m_i'(t)$ (α_{s12})	-0.10	-0.285	-0.185	0.142	0.341	0.329	91.4
**Dropout cause 2**							
Observed marker value (ϕ_{s21})	-0.02	0.006	0.026	0.001	0.029	0.028	85.3
Previous gap time (γ_{s21})	1.40	1.412	0.012	0.008	0.087	0.090	93.4
Binary covariate (γ_{s22})	0.50	0.503	0.003	0.027	0.168	0.165	95.8
$m_i(0)$ (α_{s21})	-0.05	-0.075	-0.025	0.002	0.039	0.039	91.0
$m_i'(t)$ (α_{s22})	-0.20	-0.343	-0.143	0.100	0.301	0.282	94.6

Results from 600 replications with each data set including 1000 individuals. The true marker evolution was based on a model of the form $Y_i(t) = (\beta_0 + b_{i0}) + (\beta_1 + b_{i1})\log(t+1) + (\beta_2 + b_{i2})(t/10)^3 + \epsilon_i(t)$. "True" denotes the true parameter values; "Est" the mean of the estimates over the 600 replications; "Bias" the mean bias of estimates; "MSE" the mean squared error; "ASE" the average SE, "MCSD" the empirical Monte carlo deviation of estimates and "Cov." the empirical coverage probability (%) of the confidence intervals.

Table S6. Simulation study results, where the data have been generated by the proposed model using a gap time visiting process . Fitted model (i) is correctly specified ("Correctly specified") and (ii) ignores the visiting process ("Ignoring visiting process").

Parameter	True	Est	Bias	MSE	ASE	MCSD	Cov.	Est	Bias	MSE	ASE	MCSD	Cov.
**Longitudinal**			**Correctly specified**						**Ignoring visiting process**				
Intercept (β_0)	17.20	17.235	0.035	0.146	0.395	0.381	94.6	17.189	-0.011	0.147	0.395	0.383	94.8
$\log(t+1)$ (β_1)	4.83	4.818	-0.012	0.052	0.221	0.227	94.2	4.962	0.132	0.081	0.233	0.252	88.8
$(t/10)^3$ (β_2)	-2.80	-2.787	0.013	0.571	0.711	0.756	92.7	-1.994	0.806	1.208	0.689	0.747	76.3
Mean marker value at 10 years	25.98	26.002	0.020	0.486	0.677	0.697	94.1	27.093	1.111	1.685	0.648	0.671	58.4
**Visiting process**													
Observed marker value (ϕ_{v1})	0.02	0.020	0.000	0.000	0.004	0.004	94.6						
t_{ij} (γ_{v1})	-0.02	-0.020	-0.000	0.000	0.011	0.012	94.6						
Previous gap time (γ_{v2})	-1.50	-1.502	-0.002	0.005	0.070	0.068	97.0						
$m_i(0)$ (α_{v1})	0.02	0.020	0.000	0.000	0.005	0.005	95.3						
$m_i'(t)$ (α_{v2})	0.20	0.200	0.000	0.000	0.019	0.019	96.1						
**Dropout cause 1**													
Observed marker value (ϕ_{s11})	-0.20	-0.208	-0.008	0.003	0.057	0.057	95.9	-0.215	-0.015	0.004	0.058	0.062	94.6
Binary covariate (γ_{s11})	0.50	0.561	0.061	0.164	0.378	0.400	94.8	0.567	0.067	0.177	0.385	0.415	95.3
$m_i(0)$ (α_{s11})	-0.05	-0.061	-0.011	0.006	0.076	0.076	95.6	-0.050	0.000	0.007	0.080	0.085	93.2
$m_i'(t)$ (α_{s12})	-0.10	-0.128	-0.028	0.140	0.330	0.373	92.7	-0.059	0.041	0.250	0.388	0.498	89.8
**Dropout cause 2**													
Observed marker value (ϕ_{s21})	-0.02	-0.020	-0.000	0.002	0.044	0.044	95.3	-0.029	-0.009	0.002	0.045	0.047	93.6
Previous gap time (γ_{s21})	1.40	1.448	0.048	0.045	0.197	0.206	94.1	1.453	0.053	0.048	0.199	0.212	93.6
Binary covariate (γ_{s22})	0.50	0.524	0.024	0.134	0.327	0.366	92.4	0.525	0.025	0.135	0.328	0.367	92.7
$m_i(0)$ (α_{s21})	-0.05	-0.051	-0.001	0.003	0.057	0.058	94.8	-0.035	0.015	0.005	0.062	0.067	92.4
$m_i'(t)$ (α_{s22})	-0.20	-0.213	-0.013	0.116	0.323	0.340	93.7	-0.073	0.127	0.225	0.401	0.457	89.5

Results from 600 replications with each data set including 300 individuals. The true marker evolution was based on a model of the form $Y_i(t) = (\beta_0 + b_{i0}) + (\beta_1 + b_{i1})\log(t+1) + (\beta_2 + b_{i2})(t/10)^3 + \epsilon_i(t)$. "True" denotes the true parameter values; "Est" the mean of the estimates over the 600 replications; "Bias" the mean bias of estimates; "MSE" the mean squared error; "ASE" the average SE, "MCSD" the empirical Monte carlo deviation of estimates and "Cov." the empirical coverage probability (%) of the confidence intervals.

S4.5 *Simulation study including frailties in the visiting process*

Tables S7-S10 present results from the simulation studies described in Section 3.5 of the main part of the manuscript.

Table S7. Simulation study results, where the data have been generated by the proposed model using a gap time visiting process including individual-specific frailties. Fitted model (i) is correctly specified ("Correctly specified") and (ii) ignores the visiting process ("Ignoring visiting process").

Parameter	True	Est	Bias	MSE	ASE	MCSD	Cov.	Est	Bias	MSE	ASE	MCSD	Cov.
**Longitudinal**			**Correctly specified**						**Ignoring visiting process**				
Intercept (β_0)	17.20	17.217	0.017	0.046	0.218	0.214	95.9	17.197	-0.003	0.046	0.218	0.214	96.3
$\log(t+1)$ (β_1)	4.83	4.827	-0.003	0.019	0.132	0.138	93.7	4.968	0.138	0.039	0.136	0.142	82.2
$(t/10)^3$ (β_2)	-2.80	-2.789	0.011	0.103	0.319	0.320	94.6	-2.490	0.310	0.197	0.316	0.317	84.4
Mean marker value at 10 years	25.98	26.004	0.022	0.098	0.314	0.312	95.1	26.620	0.638	0.501	0.309	0.306	45.1
**Visiting process**													
Observed marker value (ϕ_{v1})	0.02	0.020	0.000	0.000	0.003	0.005	94.1						
t_{ij} (γ_{v1})	-0.02	-0.020	-0.000	0.000	0.006	0.007	95.9						
$\log(\eta)$	-0.69	-0.694	-0.000	0.004	0.068	0.064	95.4						
$m_i(0)$ (α_{v1})	0.02	0.020	-0.000	0.000	0.005	0.009	94.2						
$m_i'(t)$ (α_{v2})	0.20	0.197	-0.003	0.002	0.013	0.050	96.8						
**Dropout cause 1**													
Observed marker value (ϕ_{s11})	-0.20	-0.204	-0.004	0.001	0.029	0.029	95.9	-0.208	-0.008	0.001	0.029	0.029	96.3
Binary covariate (γ_{s11})	0.50	0.515	0.015	0.038	0.195	0.195	94.9	0.515	0.015	0.038	0.195	0.195	94.9
$m_i(0)$ (α_{s11})	-0.05	-0.052	-0.002	0.002	0.041	0.039	96.6	-0.044	0.006	0.002	0.041	0.040	95.1
$m_i'(t)$ (α_{s12})	-0.10	-0.103	-0.003	0.040	0.193	0.201	94.9	-0.053	0.047	0.057	0.205	0.235	92.4
**Dropout cause 2**													
Observed marker value (ϕ_{s21})	-0.02	-0.020	-0.000	0.001	0.024	0.024	95.1	-0.027	-0.007	0.001	0.024	0.024	94.4
Previous gap time (γ_{s21})	1.40	1.427	0.027	0.007	0.078	0.080	93.9	1.423	0.023	0.007	0.078	0.080	93.9
Binary covariate (γ_{s22})	0.50	0.506	0.006	0.027	0.171	0.164	96.6	0.505	0.005	0.027	0.171	0.164	96.9
$m_i(0)$ (α_{s21})	-0.05	-0.049	0.001	0.001	0.033	0.034	95.3	-0.036	0.014	0.002	0.034	0.036	92.5
$m_i'(t)$ (α_{s22})	-0.20	-0.195	0.005	0.040	0.194	0.200	94.6	-0.083	0.117	0.068	0.217	0.233	89.2

Results from 600 replications with each data set including 1000 individuals. The true marker evolution was based on a model of the form $Y_i(t) = (\beta_0 + b_{i0}) + (\beta_1 + b_{i1})\log(t+1) + (\beta_2 + b_{i2})(t/10)^3 + \epsilon_i(t)$. "True" denotes the true parameter values; "Est" the mean of the estimates over the 600 replications; "Bias" the mean bias of estimates; "MSE" the mean squared error; "ASE" the average SE, "MCSD" the empirical Monte carlo deviation of estimates and "Cov." the empirical coverage probability (%) of the confidence intervals.

Table S8. Simulation study results, where the data have been generated by the proposed model under a gap time visiting process including including individual-specific frailties. Fitted models assume a simple marginal (without frailties) model for the gap time visiting process ("Simple marginal"), and current mean gap time and $N_i(t^-)$ are added to the marginal model ("Extended marginal").

Parameter	True	Est	Bias	MSE	ASE	MCSD	Cov.	Est	Bias	MSE	ASE	MCSD	Cov.
**Longitudinal**		**Simple marginal**						**Extended marginal**					
Intercept (β_0)	17.20	17.368	0.168	0.075	0.218	0.216	87.5	17.281	0.081	0.052	0.216	0.213	94.1
$\log(t+1)$ (β_1)	4.83	4.388	-0.442	0.219	0.136	0.154	12.0	4.512	-0.318	0.121	0.134	0.142	34.9
$(t/10)^3$ (β_2)	-2.80	-2.694	0.106	0.123	0.325	0.334	93.6	-2.391	0.409	0.274	0.327	0.327	76.4
Mean marker value at 10 years	25.98	25.197	-0.785	0.737	0.329	0.347	33.9	25.711	-0.271	0.172	0.315	0.315	85.9
**Visiting process**													
Observed marker value (ϕ_{v1})		0.021			0.002	0.004		0.002			0.002	0.002	
t_{ij} (γ_{v1})		0.031			0.006	0.007		-0.129			0.011	0.011	
Current mean gap time (γ_{v2})								-0.490			0.069	0.085	
$N_i(t^-)$ (slope up to 4) (γ_{v3})								0.258			0.014	0.015	
$N_i(t^-)$ (after 4) (γ_{v4})								0.071			0.004	0.004	
$m_i(0)$ (α_{v1})		0.006			0.003	0.006		0.018			0.003	0.003	
$m_i'(t)$ (α_{v2})		0.201			0.013	0.021		0.203			0.013	0.016	
**Dropout cause 1**													
Observed marker value (ϕ_{s11})	-0.20	-0.195	0.005	0.001	0.029	0.028	94.1	-0.197	0.003	0.001	0.030	0.028	94.7
Binary covariate (γ_{s11})	0.50	0.516	0.016	0.038	0.195	0.195	94.9	0.515	0.015	0.038	0.195	0.194	94.9
$m_i(0)$ (α_{s11})	-0.05	-0.068	-0.018	0.002	0.041	0.038	95.4	-0.062	-0.012	0.002	0.041	0.039	95.9
$m_i'(t)$ (α_{s12})	-0.10	-0.162	-0.062	0.037	0.177	0.183	93.4	-0.095	0.005	0.039	0.188	0.198	94.2
**Dropout cause 2**													
Observed marker value (ϕ_{s21})	-0.02	-0.012	0.008	0.001	0.024	0.024	93.2	-0.015	0.005	0.001	0.025	0.025	94.6
Previous gap time (γ_{s21})	1.40	1.432	0.032	0.007	0.078	0.080	93.2	1.432	0.032	0.007	0.078	0.080	93.1
Binary covariate (γ_{s22})	0.50	0.506	0.006	0.027	0.171	0.164	96.3	0.506	0.006	0.027	0.171	0.164	96.8
$m_i(0)$ (α_{s21})	-0.05	-0.062	-0.012	0.001	0.032	0.033	93.6	-0.055	-0.005	0.001	0.033	0.034	94.2
$m_i'(t)$ (α_{s22})	-0.20	-0.268	-0.068	0.039	0.177	0.185	92.0	-0.202	-0.002	0.040	0.191	0.201	94.6

Results from 600 replications with each data set including 1000 individuals. The true marker evolution was based on a model of the form $Y_i(t) = (\beta_0 + b_{i0}) + (\beta_1 + b_{i1})\log(t+1) + (\beta_2 + b_{i2})(t/10)^3 + \epsilon_i(t)$. "True" denotes the true parameter values; "Est" the mean of the estimates over the 600 replications; "Bias" the mean bias of estimates; "MSE" the mean squared error; "ASE" the average SE, "MCSD" the empirical Monte carlo deviation of estimates and "Cov." the empirical coverage probability (%) of the confidence intervals.

Table S9. Simulation study results, where the data have been generated by the proposed model under an intensity-based visiting process (calendar time) including individual-specific frailties. Fitted model (i) is correctly specified ("Correctly specified") and (ii) ignores the visiting process ("Ignoring visiting process")..

Parameter	True	Est	Bias	MSE	ASE	MCSD	Cov.	Est	Bias	MSE	ASE	MCSD	Cov.
**Longitudinal**			**Correctly specified**						**Ignoring visiting process**				
Intercept (β_0)	17.20	17.210	0.010	0.051	0.219	0.227	93.9	17.171	-0.029	0.053	0.218	0.228	93.2
$\log(t+1)$ (β_1)	4.83	4.829	-0.001	0.015	0.124	0.121	94.7	4.927	0.097	0.027	0.129	0.132	88.3
$(t/10)^3$ (β_2)	-2.80	-2.815	-0.015	0.094	0.301	0.307	94.4	-2.518	0.282	0.177	0.301	0.311	83.7
Mean marker value at 10 years	25.98	25.974	-0.007	0.095	0.289	0.308	92.4	26.469	0.487	0.332	0.289	0.307	59.6
**Visiting process**													
Observed marker value (ϕ_{v1})	0.02	0.020	-0.000	0.000	0.003	0.003	94.4						
$\log(\eta)$	-0.69	-0.696	-0.003	0.004	0.057	0.061	93.2						
$m_i(0)$ (α_{v1})	0.02	0.020	-0.000	0.000	0.005	0.005	95.9						
$m_i'(t)$ (α_{v2})	0.20	0.201	0.001	0.000	0.014	0.015	94.9						
**Dropout cause 1**													
Observed marker value (ϕ_{s11})	-0.20	-0.204	-0.004	0.001	0.029	0.030	96.6	-0.211	-0.011	0.001	0.029	0.030	94.9
Binary covariate (γ_{s11})	0.50	0.508	0.008	0.035	0.193	0.187	95.6	0.507	0.007	0.035	0.194	0.188	95.6
$m_i(0)$ (α_{s11})	-0.05	-0.051	-0.001	0.002	0.041	0.041	95.1	-0.033	0.017	0.002	0.041	0.044	90.0
$m_i'(t)$ (α_{s12})	-0.10	-0.096	0.004	0.040	0.184	0.201	92.0	0.024	0.124	0.083	0.207	0.261	87.8
**Dropout cause 2**													
Observed marker value (ϕ_{s21})	-0.02	-0.022	-0.002	0.000	0.022	0.022	94.7	-0.029	-0.009	0.001	0.022	0.022	92.7
Previous gap time (γ_{s21})	1.40	1.420	0.020	0.005	0.070	0.067	95.8	1.416	0.016	0.005	0.070	0.068	95.6
Binary covariate (γ_{s22})	0.50	0.510	0.010	0.028	0.169	0.167	94.7	0.510	0.010	0.028	0.169	0.168	94.7
$m_i(0)$ (α_{s21})	-0.05	-0.047	0.003	0.001	0.029	0.030	93.4	-0.034	0.016	0.001	0.030	0.031	90.3
$m_i'(t)$ (α_{s22})	-0.20	-0.172	0.028	0.039	0.193	0.195	94.6	-0.048	0.152	0.071	0.215	0.220	87.9

Results from 600 replications with each data set including 1000 individuals. The true marker evolution was based on a model of the form $Y_i(t) = (\beta_0 + b_{i0}) + (\beta_1 + b_{i1})\log(t+1) + (\beta_2 + b_{i2})(t/10)^3 + \epsilon_i(t)$. "True" denotes the true parameter values; "Est" the mean of the estimates over the 600 replications; "Bias" the mean bias of estimates; "MSE" the mean squared error; "ASE" the average SE, "MCSD" the empirical Monte carlo deviation of estimates and "Cov." the empirical coverage probability (%) of the confidence intervals.

Table S10. Simulation study results, where the data have been generated by the proposed model under an intensity-based visiting process (calendar time) including individual-specific frailties. Fitted models assume a simple marginal (without frailties) model for the intensity-based visiting process ("Simple marginal"), and current mean gap time and $N_i(t^-)$ are added to the marginal model ("Extended marginal").

Parameter	True	Est	Bias	MSE	ASE	MCSD	Cov.	Est	Bias	MSE	ASE	MCSD	Cov.
**Longitudinal**		Simple marginal						Extended marginal					
Intercept (β_0)	17.20	17.559	0.359	0.185	0.214	0.237	59.6	17.240	0.040	0.053	0.217	0.227	93.0
$\log(t+1)$ (β_1)	4.83	4.529	-0.301	0.104	0.097	0.118	16.5	4.688	-0.142	0.035	0.126	0.123	79.6
$(t/10)^3$ (β_2)	-2.80	-4.559	-1.759	3.252	0.279	0.395	0.2	-2.603	0.197	0.133	0.310	0.307	89.6
Mean marker value at 10 years	25.98	23.860	-2.122	4.667	0.309	0.405	0.0	25.878	-0.104	0.109	0.291	0.314	91.7
**Visiting process**													
Observed marker value (ϕ_{v1})		0.051			0.003	0.006		0.006			0.002	0.003	
Current mean gap time (γ_{v1})								-0.253			0.030	0.053	
$N_i(t^-)$ (slope up to 4) (γ_{v2})								0.374			0.015	0.019	
$N_i(t^-)$ (after 4) (γ_{v3})								0.040			0.001	0.004	
$m_i(0)$ (α_{v1})		-0.022			0.004	0.011		0.013			0.003	0.003	
$m_i'(t)$ (α_{v2})		0.595			0.042	0.218		0.151			0.012	0.017	
**Dropout cause 1**													
Observed marker value (ϕ_{s11})	-0.20	-0.166	0.034	0.002	0.028	0.026	75.0	-0.199	0.001	0.001	0.029	0.030	95.6
Binary covariate (γ_{s11})	0.50	0.507	0.007	0.036	0.194	0.188	95.8	0.510	0.010	0.035	0.194	0.188	95.8
$m_i(0)$ (α_{s11})	-0.05	-0.111	-0.061	0.005	0.038	0.035	66.6	-0.062	-0.012	0.002	0.041	0.042	93.9
$m_i'(t)$ (α_{s12})	-0.10	-0.268	-0.168	0.065	0.192	0.193	87.1	-0.152	-0.052	0.048	0.193	0.212	91.0
**Dropout cause 2**													
Observed marker value (ϕ_{s21})	-0.02	0.007	0.027	0.001	0.024	0.025	81.7	-0.020	-0.000	0.000	0.022	0.022	95.1
Previous gap time (γ_{s21})	1.40	1.435	0.035	0.006	0.071	0.067	93.9	1.423	0.023	0.005	0.070	0.067	95.4
Binary covariate (γ_{s22})	0.50	0.512	0.012	0.028	0.170	0.168	95.2	0.510	0.010	0.028	0.170	0.168	94.9
$m_i(0)$ (α_{s21})	-0.05	-0.087	-0.037	0.002	0.032	0.034	78.3	-0.051	-0.001	0.001	0.030	0.030	94.2
$m_i'(t)$ (α_{s22})	-0.20	-0.242	-0.042	0.046	0.214	0.210	95.8	-0.201	-0.001	0.038	0.197	0.194	95.6

Results from 600 replications with each data set including 1000 individuals. The true marker evolution was based on a model of the form $Y_i(t) = (\beta_0 + b_{i0}) + (\beta_1 + b_{i1})\log(t+1) + (\beta_2 + b_{i2})(t/10)^3 + \epsilon_i(t)$. "True" denotes the true parameter values; "Est" the mean of the estimates over the 600 replications; "Bias" the mean bias of estimates; "MSE" the mean squared error; "ASE" the average SE, "MCSD" the empirical Monte carlo deviation of estimates and "Cov." the empirical coverage probability (%) of the confidence intervals.

S5. Additional results from the application to data from the AMACS study

In this section, we consider a basic exploratory analysis as described in detail in Cook and Lawless (2007). Specifically, in a recurrent-event analysis with intensity function $\lambda_i(t)$, the generalized residuals for the ith individual are defined as

$$E_{ij} = \int_{T_{i,j-1}}^{T_{ij}} \lambda_i(u)du, \quad j = 1, 2, \ldots,$$

where T_{ij}, $j = 1, 2, \ldots$, represent the visit times for the ith individual. If the model is correct, the generalized residuals E_{ij} should be distributed as standard (rate=1) exponential random variables. In practice, $\lambda_i(t)$ is replaced by the respective maximum likelihood estimate, and T_{ij} by their corresponding realizations t_{ij}. This approach is similar to the Cox-Snell residuals, frequently applied in standard survival analysis.

As the last visit time for each individual is typically right-censored, we computed the Nelson-Aalen estimate of the generalized residuals. The plot of this estimate should form a line with slope 1 (the cumulative hazard function of the standard exponential distribution). We computed the Nelson-Aalen estimate of the generalized residuals, with the random effects equal to their empirical Bayes estimates, for both the gap time model and the intensity-based model using calendar time fitted to the AMACS data. The results are presented in Figure S3.

This plot clearly suggests that the gap time model has a substantially better fit compared to the intensity-based model using calendar time, although the fitted gap time model may not perfectly represent the true model. This informal diagnostic plot confirms and supports our previous findings, as the gap time model has a substantially lower AIC compared to the calendar time model.

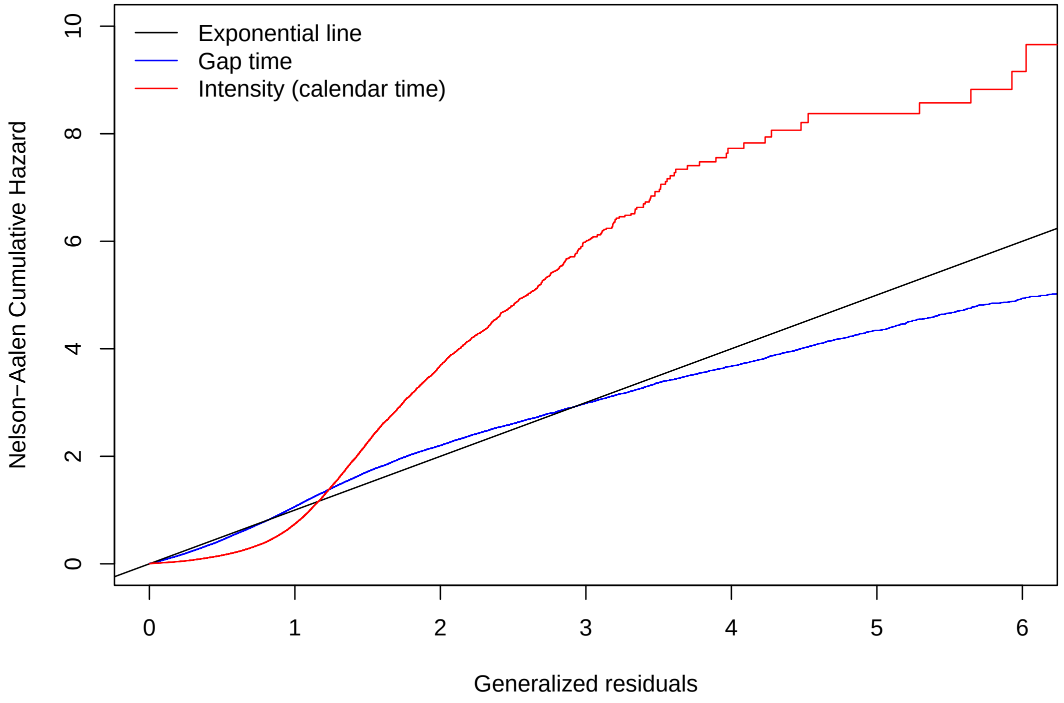

Fig. S3. Generalized residuals for the visiting process for the gap-time model and the intensity-based model using calendar time fitted to data from the AMACS study.

Table S11. Results from the proposed joint model fitted to data from the Athens Multicenter AIDS Cohort Study (AMACS).

**Parameter**	**Estimate**	**95% CI**	**p**
Intercept	16.533	(15.796,17.269)	< 0.001
Natural splines-1 (MSM)	6.519	(6.26,6.777)	< 0.001
Natural splines-2 (MSM)	7.073	(6.758,7.389)	< 0.001
Natural splines-3 (MSM)	14.286	(13.863,14.709)	< 0.001
Natural splines-4 (MSM)	6.080	(5.706,6.455)	< 0.001
Natural splines-1 (PWID/MSM)	-2.291	(-3.012,-1.569)	< 0.001
Natural splines-2 (PWID/MSM)	-0.791	(-1.778,0.195)	0.116
Natural splines-3 (PWID/MSM)	-3.264	(-4.453,-2.076)	< 0.001
Natural splines-4 (PWID/MSM)	-0.639	(-2.102,0.824)	0.392
PWID/MSM	-2.923	(-3.592,-2.253)	< 0.001
Age at ART initiation (yrs)	-0.115	(-0.144,-0.086)	< 0.001
Prior AIDS	-5.590	(-6.301,-4.879)	< 0.001
Year of HIV diagnosis	0.237	(0.19,0.284)	< 0.001

Presented results refer to the parameters of the CD4 model using a gap-time visiting process using frailties. Corresponding results for the competing risks have been presented in Table 3 of the main part of the manuscript.

Recall that the random-effect structure was based on natural splines with 2 internal knots at 1 and 5 years since ART initiation. The estimated variance-covariance matrix of the random effects is

$$\hat{\boldsymbol{D}} = \begin{pmatrix} 31.82 & -7.72 & -16.22 & -7.85 \\ -7.72 & 23.11 & 18.54 & 12.01 \\ -16.22 & 18.54 & 51.92 & 18.52 \\ -7.85 & 12.01 & 18.52 & 20.24 \end{pmatrix}$$

and the estimated within-individual variance $\hat{\sigma}^2 = 7.31$. The estimated correlations between the random effects range from -0.40 to 0.57.

S6. Results from one simulated dataset

The following results were obtained using the code posted as supplementary material available at http://biostatistics.oxfordjournals.org [R version 4.3.2 (2023-10-31 ucrt)]

```
##############################################
### Fit the proposed model using gap times ###
##############################################
fitPropGap <- PropJMvisDropCompMLE(fitlme,fitCoxDrop1,fitCoxDrop2,fitCoxVisGap,
                                   nknotsDrop1 = 1,nknotsDrop2 = 1,nknotsVis = 3,nGH = 4)


> round(fitPropGap$sumLong,3)
                  Estimate    SE     LB     UB
(Intercept)         17.509 0.217 17.085 17.934
I(log(times + 1))    5.004 0.120  4.769  5.239
I((times/10)^3)     -2.876 0.370 -3.602 -2.151
log(sigma^2)         2.090 0.014  2.063  2.118
Vech(logD)1          3.702 0.048  3.608  3.796
Vech(logD)2         -0.466 0.038 -0.540 -0.392
```

```
Vech(logD)3         -0.157 0.062 -0.278 -0.036

Vech(logD)4          1.596 0.064  1.470  1.722

Vech(logD)5         -0.521 0.057 -0.633 -0.410

Vech(logD)6          3.352 0.105  3.146  3.557

> round(fitPropGap$sumVis,3)

          Estimate    SE     LB     UB

y            0.020 0.002  0.016  0.025

times       -0.019 0.006 -0.031 -0.007

lag1_gap    -1.505 0.037 -1.579 -1.432

AssocVis1    0.019 0.002  0.014  0.024

AssocVis2    0.199 0.010  0.179  0.219

PsiVis1     -5.980 0.242 -6.454 -5.507

PsiVis2     -0.500 0.117 -0.730 -0.270

PsiVis3     -0.039 0.073 -0.183  0.105

PsiVis4      0.665 0.056  0.556  0.775

PsiVis5     -0.699 0.219 -1.128 -0.270

PsiVis6     -7.063 0.939 -8.904 -5.223

PsiVis7     -2.954 1.924 -6.725  0.817

> round(fitPropGap$sumDrop1,3)

            Estimate    SE     LB     UB

y             -0.186 0.031 -0.248 -0.125

group          0.753 0.207  0.347  1.159

AssocDrop11   -0.024 0.041 -0.103  0.056

AssocDrop12    0.140 0.159 -0.171  0.452

PsiDrop11     -1.269 1.355 -3.925  1.386
```

```
PsiDrop12     -1.148 0.756 -2.630  0.334
PsiDrop13     -0.912 0.921 -2.717  0.892
PsiDrop14     -1.292 1.125 -3.496  0.912
PsiDrop15     -1.261 0.750 -2.731  0.210
> round(fitPropGap$sumDrop2,3)
            Estimate    SE     LB     UB
y             -0.021 0.026 -0.072  0.030
lag1_gap       1.349 0.107  1.141  1.558
group          0.527 0.180  0.175  0.879
AssocDrop21   -0.058 0.033 -0.123  0.007
AssocDrop22   -0.131 0.171 -0.467  0.205
PsiDrop21     -1.643 1.019 -3.640  0.354
PsiDrop22     -1.920 0.681 -3.256 -0.585
PsiDrop23     -4.146 0.789 -5.692 -2.601
PsiDrop24     -6.452 1.019 -8.449 -4.456
PsiDrop25     -6.449 0.788 -7.994 -4.905


##################################################
### Fit the proposed model using calendar time ###
##################################################
fitCoxVisVcal <- coxph(Surv(tstart,tstop,deltaVis) ~ y + lag1_gap + cluster(id),
                        data = dataLong,control = coxph.control(timefix = FALSE))
summary(fitCoxVisVcal)
```

```
# Fit the proposed model

fitPropCal <- PropJMvisDropCompMLE(fitlme, fitCoxDrop1,fitCoxDrop2, fitCoxVisVcal,
                                   nknotsDrop1 = 1,nknotsDrop2 = 1,nknotsVis = 3,nGH = 4)


> round(fitPropCal$sumLong,3)
                  Estimate    SE     LB     UB
(Intercept)         17.441 0.218 17.014 17.868
I(log(times + 1))    5.180 0.122  4.941  5.420
I((times/10)^3)     -3.401 0.417 -4.219 -2.583
log(sigma^2)         2.089 0.014  2.062  2.116
Vech(logD)1          3.719 0.047  3.627  3.811
Vech(logD)2         -0.474 0.038 -0.548 -0.399
Vech(logD)3         -0.045 0.064 -0.170  0.079
Vech(logD)4          1.602 0.064  1.476  1.727
Vech(logD)5         -0.449 0.060 -0.567 -0.332
Vech(logD)6          3.426 0.111  3.208  3.644
> round(fitPropCal$sumVis,3)
          Estimate    SE     LB     UB
y            0.009 0.002  0.005  0.014
lag1_gap    -1.593 0.036 -1.664 -1.522
AssocVis1    0.019 0.003  0.013  0.024
AssocVis2    0.115 0.012  0.091  0.139
PsiVis1     -0.910 0.107 -1.120 -0.701
PsiVis2      0.681 0.081  0.523  0.839
PsiVis3      0.154 0.071  0.016  0.293
```

```
PsiVis4      0.472 0.066  0.342  0.602

PsiVis5      0.165 0.088 -0.007  0.337

PsiVis6      0.181 0.100 -0.015  0.376

PsiVis7     -0.079 0.090 -0.254  0.097

> round(fitPropCal$sumDrop1,3)

            Estimate    SE     LB     UB

y             -0.197 0.032 -0.259 -0.135

group          0.748 0.208  0.341  1.156

AssocDrop11   -0.005 0.042 -0.086  0.077

AssocDrop12    0.196 0.183 -0.163  0.555

PsiDrop11     -1.850 1.660 -5.104  1.404

PsiDrop12     -1.460 0.881 -3.187  0.268

PsiDrop13     -0.901 0.923 -2.710  0.908

PsiDrop14     -1.402 1.135 -3.626  0.822

PsiDrop15     -1.237 0.749 -2.704  0.230

> round(fitPropCal$sumDrop2,3)

            Estimate    SE     LB     UB

y             -0.029 0.026 -0.081  0.023

lag1_gap       1.339 0.105  1.134  1.544

group          0.529 0.180  0.177  0.882

AssocDrop21   -0.045 0.035 -0.113  0.023

AssocDrop22   -0.047 0.191 -0.422  0.328

PsiDrop21     -2.125 1.259 -4.592  0.342

PsiDrop22     -2.201 0.774 -3.718 -0.684

PsiDrop23     -4.156 0.792 -5.708 -2.605
```

```
PsiDrop24     -6.560 1.027 -8.572 -4.548

PsiDrop25     -6.389 0.777 -7.912 -4.865
```

References

Cook, R.J. and Lawless, J. (2007). *The Statistical Analysis of Recurrent Events*, Statistics for Biology and Health. Springer New York.

Duchateau, L. and Janssen, P. (2007). *The Frailty Model*, Statistics for Biology and Health. Springer New York.

Ryu, Duchwan, Sinha, Debajyoti, Mallick, Bani, Lipsitz, Stuart R. and Lipshultz, Steven E. (2007). Longitudinal studies with outcome-dependent follow-up: Models and bayesian regression. *Journal of the American Statistical Association* **102**(479), 952–961.

Thomadakis, Christos, Meligkotsidou, Loukia, Pantazis, Nikos and Touloumi, Giota. (2020). Misspecifying the covariance structure in a linear mixed model under mar drop-out. *Statistics in Medicine* **39**(23), 3027–3041.
